# Silicate solubilizing and plant growth promoting bacteria interact with biogenic silica to impart heat stress tolerance in rice by modulating physiology and gene expression

**DOI:** 10.3389/fmicb.2023.1168415

**Published:** 2023-07-13

**Authors:** Chandrakala Chaganti, Amol Sarjerao Phule, Latha P. Chandran, Bandeppa Sonth, Venkat Prasad Babu Kavuru, Rajani Govindannagari, Raman Meenakshi Sundaram

**Affiliations:** ICAR-Indian Institute of Rice Research, Hyderabad, Telangana, India

**Keywords:** heat stress, silicon, plant growth promoting bacteria, *Gluconacetobacter diazotrophicus*, *Rhizobium* sp. IIRR N1, gene expression, qRT-PCR, rice

## Abstract

Heat stress caused due to increasing warming climate has become a severe threat to global food production including rice. Silicon plays a major role in improving growth and productivity of rice by aiding in alleviating heat stress in rice. Soil silicon is only sparingly available to the crops can be made available by silicate solubilizing and plant-growth-promoting bacteria that possess the capacity to solubilize insoluble silicates can increase the availability of soluble silicates in the soil. In addition, plant growth promoting bacteria are known to enhance the tolerance to abiotic stresses of plants, by affecting the biochemical and physiological characteristics of plants. The present study is intended to understand the role of beneficial bacteria viz. *Rhizobium* sp. IIRR N1 a silicate solublizer and *Gluconacetobacter diazotrophicus*, a plant growth promoting bacteria and their interaction with insoluble silicate sources on morpho-physiological and molecular attributes of rice (*Oryza sativa* L.) seedlings after exposure to heat stress in a controlled hydroponic system. Joint inoculation of silicates and both the bacteria increased silicon content in rice tissue, root and shoot biomass, significantly increased the antioxidant enzyme activities (viz. superoxidase dismutase, catalase and ascorbate peroxidase) compared to other treatments with sole application of either silicon or bacteria. The physiological traits (viz. chlorophyll content, relative water content) were also found to be significantly enhanced in presence of silicates and both the bacteria after exposure to heat stress conditions. Expression profiling of shoot and root tissues of rice seedlings revealed that seedlings grown in the presence of silicates and both the bacteria exhibited higher expression of heat shock proteins (HSPs viz., *OsHsp90*, OsHsp100 and *60 kDa chaperonin*), hormone-related genes (*OsIAA6*) and silicon transporters (*OsLsi1* and *OsLsi2*) as compared to seedlings treated with either silicates or with the bacteria alone. The results thus reveal the interactive effect of combined application of silicates along with bacteria *Rhizobium* sp. IIRR N1, *G. diazotrophicus* inoculation not only led to augmented silicon uptake by rice seedlings but also influenced the plant biomass and elicited higher expression of HSPs, hormone-related and silicon transporter genes leading to improved tolerance of seedling to heat stress.

## Introduction

Global warming is predicted to become a serious threat to the food security of many countries (Xu et al., 2021) as the resultant heat stress is a major constraint to crop productivity worldwide (Janni et al., 2020). Heat stress is an increase in temperature above a threshold level for a certain period that causes an irreversible effect on the growth and development of plants at all stages, i.e., from germination to harvesting (Govindaraj et al., 2018; Ding et al., 2021; Ali et al., 2022; Ejaz et al., 2022). Every 1°C increase in global mean temperature has been predicted to decrease global yields of rice by 3.2%, maize by 7.4%, wheat by 6.0%, and soybean by 3.1% (Zhao et al., 2017).

Rice (*Oryza sativa L*.) is a major food crop for more than half of the world’s population. The adverse effects of heat stress are estimated to reduce rice production by 41% by the end of the 21st century (Shi et al., 2017). Rice crop maintains normal growth at temperatures ranging from 27 to 32°C without any significant reduction in yield, but temperatures above 32°C negatively affect rice plant development at all growth stages (Aghamolki et al., 2014). The early growth stage is one of the most vital stages of growth, playing an important role in stand establishment that is crucial for realizing rice yield potential (Finch-Savage and Bassel, 2016). This stage of rice is highly susceptible as seedlings experience more heat stress due to their smaller sizes and high radiative energy from the soil and water interface (Jagadish et al., 2021). The optimum growth temperature of rice at the seedling stage is 25–28°C. Heat stress (42–45°C) at the seedling stage, results in increased water loss, withered and yellow leaves, impaired seedling and root growth, and even death of seedlings (Xu et al., 2021).

Heat stress deleteriously alters the morphological (growth and plant biomass), anatomical, and physio-biochemical characteristics (stability of cell membranes and proteins, photosynthetic apparatus, and carbohydrate metabolism) of plants (Ul Hassan et al., 2021; Xu et al., 2021) which can, in case of rice, result in a significant qualitative and quantitative reduction in grain and straw yield. At a molecular level, following plants’ sensing of heat stress, several signal cascades are induced, activating transcriptional responses relating to reactive oxygen species (ROS) regulation, plant hormones (Devireddy et al., 2021) and other molecular components like heat shock proteins. ROS, as key signaling molecules, play a pivotal role in enabling plant cells to respond rapidly to heat stress (Mittler et al., 2022). Heat stress disrupts redox homeostasis, which exacerbates ROS toxicity by inactivating antioxidant enzymes, including ascorbate peroxidase (APX), catalase (CAT), glutathione peroxidase (GPX), superoxide dismutase (SOD), and glutathione S-transferase (GST) and decreasing the synthesis of non-enzymatic antioxidants such as ascorbic acid, glutathione and phenolic compounds (Sharma et al., 2019; Hasanuzzaman et al., 2020). Phytohormones such as ethylene, auxins, cytokinins, abscisic acid, salicylic acid, jasmonates, brassinosteroids and strigalactones are another group of signaling molecules which are endogenous and help in mediating plant response to stresses, including heat stress (Li et al., 2021). Another molecular response is the expression of heat stress and other related proteins that act as molecular chaperones protecting plants from proteotoxic stress caused by protein misfolding and denaturation (Xu et al., 2021). Tolerance to heat stress is conferred by maintaining plant functions and efficient scavenging of ROS (Lei et al., 2018) and stabilizing the function and structure of protein and enzymes by heat shock proteins (HSPs) in plants (Maestri et al., 2002). An integrated approach using multiple techniques like crop (genetic improvement) and soil management (agronomic) strategies is necessary (Zafar et al., 2018) for imparting thermotolerance in crops. From an agronomic perspective, silicon (Kumar et al., 2022) and beneficial microbe based technologies (Shekhawat et al., 2022) are currently receiving considerable interest as they can be harnessed to provide eco-friendly and cost effective stress mitigating solutions for sustainable heat tolerant crop production systems.

Silicon (Si) is a quasi-essential and beneficial nutrient element involved in the growth and development of crops (Epstein, 1999; Ma et al., 2001; Liang et al., 2005; Hamayun et al., 2010; Kim et al., 2011), particularly under various stresses. Under abiotic stress conditions (Farooq and Dietz, 2015; Muneer et al., 2017; Rahman et al., 2017; Hussain et al., 2019; Zargar et al., 2019; Younis et al., 2020), Si acts as a stress alleviator by enhancing membrane integrity and antioxidant defense system through alterations in cellular and biochemical mechanisms. The role of Si in inducing heat stress tolerance in plants (Tripathi et al., 2017; Frew et al., 2018; Saha et al., 2021; Kumar et al., 2022) including agriculturally important crops like rice is well known (Jinger et al., 2020). In rice, tissue Si plays a major role in the growth and productivity of rice through increases in the strength of stems, erectness of leaves, and photosynthetic rate (Berahim et al., 2021) alleviating injurious effect of stresses in rice (Etesami and Jeong, 2018; Mostofa et al., 2021). In rice, Si is absorbed by rice roots in the form of silicic acid through Si influx (Lsi1) and efflux transporters (Lsi2) (Ma et al., 2011; Yamaji et al., 2015). Further, Si in soil solution taken up by rice roots is translocated into shoots through the transpiration stream of the xylem and unloaded by Lsi6 transporter (Yamaji et al., 2015) where it ultimately accumulates under the cuticle and intracellular spaces (Heckman, 2013) and precipitates in plant cells as phytoliths (Cooke and Leishman, 2011; Rao and Susmitha, 2017).

Since Si plays a prophylactic role under stress conditions, root and shoot tissues, especially in monocots like rice, must possess a minimum concentration of Si to combat the negative effect of stresses (Debona et al., 2017). Additionally, soluble Si only when taken up through the root system and transported from roots via the xylem alone, can activate the metabolic processes that induce beneficial changes in plants (Zellener et al., 2021). Hence the benefits of Si are realizable only when Si, present abundantly in soil and parent mineral rocks as insoluble silicates (Haynes, 2017), is transformed into plant-available soluble Si. Geochemical weathering of silicates, during which dissolved Si in the form of plant-available silicic acid (H_4_SiO_4_) is released, is inadequate to replenish soluble Si to meet the crop needs as it is an inconsistent process occurring over extended periods (Rao et al., 2019). Soluble Si release from parent rocks also declines with soil age in countries with mature soils (Madhuri, 2018) like India due to desilication which occurs continuously over time. Nutrient stripping when growing silicon-accumulating crops like rice, where the average content of Si is usually more than 40 mg/g of dry biomass (Hughes et al., 2020), also aggravates Si depletion in soil. Si-containing fertilizers can mitigate this loss by increasing the bioavailability of soil Si and fulfilling the Si demand for crops. However, though different commercially produced Si fertilizers are available (Tayade et al., 2022), their use is often constrained by the costs, efficacy and ease of handling and application, besides encountering conflicts in identifying the appropriate source of fertilizer (Haneklaus et al., 2018). For improving soil bioavailable Si concentrations, the newer and smarter silicon fertilization strategies that are currently under reckoning due to their environment-friendly, agronomically efficient, and renewable nature are (i) the use of biogenic siliceous material as Si source and (ii) soil inoculation with silicate solubilizing microorganisms (Meena et al., 2014; Constantinescu-Aruxandei et al., 2020; Hughes et al., 2020; Raturi et al., 2021). An optimal combination of biogenic silica along with silicate solubilizing bacteria can lead to additive effects by the release of soluble Si from amorphous insoluble Si present in rice straw and diatomaceous earth. However, there are very few studies related to phytogenic/biogenic Si solubilization by microorganisms (Constantinescu-Aruxandei et al., 2020).

Rhizospheric and endophytic plant-growth promoting microorganisms (PGPM) can colonize plant roots (rhizosphere) and increase plant growth (shoot and root growth) and yield through various mechanisms like biosynthesis of phytohormones, exopolysaccharides, and volatile organic compounds (Adhikari et al., 2020; Kim et al., 2020) in addition to stimulating enzymatic activity, production of antioxidants and osmoprotectants (Wang et al., 2018). PGPMs enhance plant tolerance to abiotic stresses, by affecting the biochemical and physiological characteristics of plants (Etesami and Maheshwari, 2018; Asghari et al., 2020). There is increasing evidence that heat stress responses and recovery of plants also depends on their interaction with different microorganisms (Chandrakala et al., 2019a; Dastogeer et al., 2022). While plant growth promotion and stress-alleviating traits of rhizospheric microorganisms have been widely recognized, investigations are still ongoing to understand the endophytic micro-organisms and their role as stress controllers in plants (Mahapatra, 2017; Lata et al., 2018). Navarro-Torre et al. (2023), have put forward the theory that colonization by endophytic bacteria with the ability for vertical transmission across plant generations, can make plants constitutively thermotolerant, thus becoming a potent tool in the management of heat stress in plants. Beneficial microbe-mediated plant thermotolerance is an ecologically sustainable strategy to mitigate heat stress in plants (Shekhawat et al., 2022) but only a few studies have documented interactions between plants and endophytic microbes in the control of heat stress (Shaffique et al., 2022).

Co-application of insoluble silicates along with multiple plant growth promoting bacteria is expected to benefit heat-stressed plants (Al-Garni et al., 2019; Chandrakala et al., 2019b; Vishwakarma et al., 2020), however, research on the synergistic effects due to combined application of siliceous material along with microbes for heat stress alleviation has been scarce. With this background, the present study aimed to understand the interactive effect of the combined application of silicate solubilizing bacteria (*Rhizobium* sp. IIRR N1), plant growth promoting endophytic bacteria (*G. diazotrophicus*), and insoluble silicate sources (diatomaceous earth and rice straw), on morpho-physiological and molecular effects on rice (*Oryza sativa L*.) seedlings exposed to heat stress under a controlled hydroponic system.

## Materials and methods

### Plant genotype, siliceous material, and bacteria

Surface sterilized and germinated rice seedlings (cv. Swarna) a heat susceptible genotype (Chandel et al., 2013; Umesh et al., 2016) were grown aseptically in a hydroponics assembly. The assembly consisted of phyta jars containing 150 mL Yoshida medium (Yoshida et al., 1972) and perforated glass stand inserts on which germinated seedlings (6 seedlings) were supported such that the roots were immersed in the liquid medium (Chandrakala et al., 2019a). The Yoshida medium was amended with biogenic insoluble silicates sources [diatomaceous earth – 0.01 gm (Himedia RM 3380), which is equivalent to 75 kg/ha and rice straw – 0.33 gm equivalent to 150 kg/ha] and inoculated with bacterial cultures (1× 10^8^ CFU/mL) of *Rhizobium* sp. IIRR N1 (Accession No. KY348774) and *G. diazotrophicus* PAL 5 (MTCC 1224 acquired from CSIR – Institute of Microbial Technology, Chandigarh). Four such phyta jars with seedlings were placed in a transparent autoclavable bag with a 0.02 μm gas exchange filter patch (Sunbags, Sigma-Aldrich, Cat. no. B7026) that enabled sterile air exchange into the assembly and sealed. The rice seedlings were grown for 30 days under 16 h light and 8 h dark cycles at ambient temperature (28 ± 2°C). After 30 days of co-culturing seedlings in the presence of bacteria and insoluble silicates, the seedlings were subjected to two temperature regimes, i.e., ambient temperature (non-stressed condition) and elevated temperature (heat stress conditions). The seedlings were exposed to elevated temperature stress (45 ± 2°C) for 24 h in a growth chamber after which the seedlings were maintained under ambient conditions for 24 h (Sarsu et al., 2018). The non-stressed plants continued to grow under ambient temperature (28 ± 2°C) conditions during this 48 h period. Parallelly, controls were maintained by growing seedlings in the absence of silicate sources and bacteria. There were hence, four treatments under ambient conditions and four under heat stress. The treatments under ambient temperature/heat stress conditions were (i) seedlings grown in the absence of silicates and bacteria (AC/HS), (ii) seedlings co-cultured with both bacteria (AC+ RG /HS+ RG), (iii) seedlings co-cultured with insoluble diatomaceous earth and straw (AC+ Si/HS + Si), and (iv) seedlings co-cultured with insoluble diatomaceous earth and straw and bacteria (AC+ Si + RG/HS+ Si + RG). Each sunbag with 4 phytajars constituted a replication and three replications were maintained for each treatment. Twenty-four hours after heat stress treatment, the seedlings along with the respective controls were collected for analyzing silica uptake, morphological, physiological, biochemical and molecular response of rice seedlings to bacterial inoculation in the presence of insoluble silicates.

### Morphological characterization of seedlings

Morphological parameters *viz*. root and shoot biomass (g) of single rice seedlings were recorded manually in three replications (each replication consisted of three seedlings) under ambient and heat stress conditions after oven drying the seedlings at 60°C for 72 h.

### Silicon content in rice seedlings

Silicon content in the root and shoot of rice seedlings was determined by the colorimetric silico-molybdate blue method after autoclave-induced digestion of the samples as described by Elliott and Synder (1991).

### Physiological and biochemical characterization of seedlings

#### Chlorophyll content

The method of Zhang et al. (2009) was used for quantification of chlorophyll content in seedling leaves. Leaf sample (100 mg) was added to 25 mL of 80% acetone and after incubation for 48 h in dark at ambient temperature, the absorption of the supernatant was measured at 663 and 645 nm using a spectrophotometer (UV 1800, Shimadzu, Japan) and the chlorophyll content was calculated by using equations Arnon’s (1949).

#### Electrolyte leakage (EC)

Leaf samples from treated and untreated seedlings were cut in to uniform pieces and placed in a beaker containing 10 mL of distilled water and incubated for 4 h in a shaking incubator (Gyromax 787R, Amerex Instruments; Lafeyette, CA, United States) at room temperature. After 4 h, ion leakage (E1) was measured using conductivity meter (ECTestr 11+, Eutech Instruments, Oakton, United States). The samples were then autoclaved for 15 min and after cooling and total ion leakage (E2) was measured. EC was calculated (Jambunathan, 2010) as: EC = E1/E2 * 100.

#### Relative water content (RWC)

Leaves of all samples were cut and weighed immediately for fresh weight (FW) and immersed in water for 4 h. After immersion, excess water was removed from the leaves by blotting and then weighed to get turgid weight (TW). Leaves were oven dried at 80°C for 72 h, dry weight (DW) of the samples were recorded and the RWC was determined (Nayyar et al., 2014) using the equation: RWC (%) = (FW-DW/TW-DW) * 100.

#### Antioxidant enzyme activities

Leaf sample (200 mg) was weighed and ground into fine powder with liquid nitrogen by using pre cooled mortar and pestle. The exact weight of each sample (powdered) was determined before it was homogenized thoroughly in 1.2 mL of potassium phosphate buffer (0.2 M), pH – 7.8 with EDTA (0.1 mM). After homogenization, samples were centrifuged at 15,000 rpm for 20 min at 4°C and the supernatant was collected in a 2 mL eppendorf tube. The pellet was extracted again in 0.8 mL of the same buffer, and the combined supernatants were collected and stored in ice. This crude extract was used to determine antioxidant enzyme activities (Elavarthi and Martin, 2010).

#### Superoxide dismutase

The modified NBT method was used to determine superoxide dismutase activity. The reaction mixture (2 mL) was prepared by using 50 mM phosphate buffer (pH 7.8) with 2 mM EDTA, 9.9 mM L-methionine, 55 μM NBT and 0.025% Triton – X 100; to this 2 times diluted 40 μL of crude extract and 20 μL of riboflavin (1 mM) were added. Two sets of samples were prepared, one set was exposed to 15 W fluorescent tube, while another set was kept in the dark, and both the sets were incubated for 10 min. Samples kept in the dark were used as blanks. The absorbance of the reaction mixture was read at 560 nm (UV 1800, Shimadzu, Japan) immediately after incubation against a standard curve obtained from pure SOD (Beyer and Fridovich, 1987) and used for estimating the SOD activity.

#### Catalase

Hydrogen peroxide (10 mM, 1 mL) was added to 2 mL of 200 times diluted crude extract in 50 mM potassium phosphate buffer (pH-7), and the absorbance was read immediately at 240 nm in a UV/Vis spectrophotometer (UV 1800, Shimadzu, Japan). Decomposition of hydrogen peroxide (H_2_O_2_) was determined by using the extinction coefficient of H_2_O_2_ (43.6 M^−1^ cm^−1^ at 240 nm) and the catalase activity is expressed as mM H_2_O_2_ per minute per mg protein (Aebi and Lester, 1984).

#### Ascorbate peroxidase

Crude extract (10 μL) was added to 1 mL of assay mixture (50 mM potassium phosphate buffer (pH-7), 0.5 mM ascorbate, 0.5 mM H_2_O_2_) and the decrease in absorbance due to oxidation of ascorbate was read at 290 nm (UV 1800, Shimadzu, Japan). The extinction coefficient of 2.8 mM^−1^ cm^−1^ was used to calculate the enzyme activity which is expressed as milli mole of ascorbate per minute per mg protein (Nakano and Asada, 1981).

#### Glutathione reductase (GR)

Assay mixture (1 mL) was prepared by adding 10 μL of crude extract, 0.75 mM of 5–5′-Dithiobis (2-nitrobenzoic acid), 0.1 mM NADPH and 1 mM oxidized glutathione (GSSG). GSSG was added last to initiate the reaction and the increase in absorbance was read at 412 nm for 3 min. The extinction coefficient of 2-nitro-5-thiobenzoate (14.15 M^−1^ cm^−1^) was used to calculate GR activity and expressed as mM TNB/min/gram fresh weight (Smith et al., 1988).

#### Protein content in leaf extract

The leaf extract which was used for determining antioxidant activities was used for estimating total protein content by using Lowry’s method (Lowry et al., 1951).

### RNA isolation, cDNA synthesis, and gene expression analysis

Total RNA was isolated from the root and shoot tissue of seedlings grown under ambient and heat-stressed conditions using NucleoSpin RNA plant kit (Macherey-Nagel, Germany) following the manufacturer’s protocol. The quality and quantity of the pooled RNA (3 replications together) of each sample were checked using agarose gel electrophoresis (1%, w/v) and NanoDrop ND-1000 spectrophotometer (Thermo Fisher Scientific, Waltham, MA, United States). Expression profiling of genes related to heat shock proteins (*viz. OsHsp 26.7*, *OsHsp 70*, *OsHsp 90 OsHsp 100*, and *60 kDa chaperone*), an auxin-responsive protein (*OsIAA6*), an ethylene response factor (*OsERF1*) and three silicon transporters (*OsLsi1*, *OsLsi2* and *OsLsi6*) were studied by using qRT-PCR analysis. The qRT-PCR primers for the selected genes were designed online by using QuantPrime high throughput qRT-PCR tool^1^ by considering default parameters (Supplementary Table S1). The cDNA synthesis and qRT-PCR were performed as previously described by Phule et al. (2018, 2019). The expression levels of genes in the shoot and root tissues under ambient and heat stressed conditions were calculated using the 2-ΔΔCT method (Livak and Schmittgen, 2001) and heatmaps were plotted using MeV (v4.9.0, Multi Experiment Viewer) software (Saeed et al., 2003).

### Data analysis

Stress tolerance index (Fernandez, 1992) of the seedling biomass trait, was computed using iPASTIC online tool kit (Pour-Aboughadareh et al., 2019) to identify the best performing treatments. All recorded data were analyzed by using the statistical tool (Statistix 8.1 v2.0.1) by calculating Analysis of Variance (ANOVA) and significant difference among the treatment means was determined using least significant differences (LSD) test at 5% probability level (*p* ≤ 0.05). Pearson’s correlations between various measured traits were derived using Microsoft Excel and the corellogram was plotted using the corrplot function of the R statistical package v.3.6.0.

## Results

### Seedling biomass and tissue silica content

The bacterial (*Rhizobium* sp. IIRR N1 plus *Gluconacetobacter diazotrophicus*) and silicate treatments were generally effective in enhancing seedling biomass (shoot and root weight) and tissue silicon (Si) content in relation to untreated control at both ambient (28 ± 2°C) and high temperature (45 ± 2°C), even as heat stress modulation was observed in all treatments (Table 1).

**Table 1:** Seedling biomass and tissue silica content under ambient and heat stress conditions.

Treatments	Seedling biomass (g)*		Tissue silica content (μg/mg)*	
	Shoot	Root	Shoot	Root
Ambient (28 ± 2°C)				
AC	0.15	0.13	56.0	36.8
AC + RG	0.16	0.17	58.2	39.1
AC + Si	0.20	0.17	61.3	42.0
AC + Si + RG	0.22	0.19	66.4	45.7
Mean	0.18	0.17	60.5	40.9
CD (*P* < 0.05)	0.02	0.04	2.1	2.5
CV (%)	13.2	12.1	4.2	7.4
Heat stress (45 ± 2°C)				
HS	0.09	0.10	46.9	27.1
HS + RG	0.12	0.14	48.2	30.8
HS + Si	0.13	0.11	59.1	34.8
HS + Si + RG	0.15	0.18	63.7	39.0
Mean	0.12	0.13	54.5	32.9
CD (*P* < 0.05)	0.01	0.03	2.4	2.9
CV (%)	17.3	18.7	5.3	10.7

* Dry weight.

AC, Seedlings grown in the absence of silicates and bacteria under ambient temperature.

AC + RG, Seedlings co-cultured with *Rhizobium* sp. IIRR N1 plus *G. diazotrophicus* bacteria under ambient temperature.

AC + Si, Seedlings co-cultured with solely with insoluble diatomaceous earth and straw under ambient temperature.

AC + Si + RG, Seedlings co-cultured with both silicon and *Rhizobium* sp. IIRR N1 plus *G. diazotrophicus* under ambient temperature.

HS, Seedlings grown in the absence of silicates and bacteria under heat stress condition.

HS + RG, Seedlings co-cultured with *Rhizobium* sp. IIRR N1 plus *G. diazotrophicus* bacteria under heat stress.

HS + Si, Seedlings co-cultured with solely with insoluble diatomaceous earth and straw under heat stress.

HS + Si + RG, Seedlings co-cultured with both silicon and *Rhizobium* sp. IIRR N1 plus *G. diazotrophicus* under heat stress.

Shoot biomass showed significant changes in response to bacterial inoculation, growth in silicate sources and high temperature. At ambient temperature relative to control (AC), bacterial inoculation improved the shoot biomass by 47 and 7% in the presence (AC + Si + RG) and absence of insoluble silicates (AC + RG). Seedlings grown solely with insoluble silicates (AC + Si) also increased root biomass by 33% more than control. Similarly, post heat stress, shoot biomass was higher by 32, 62, and 44%, respectively over control (HC) in HS + RG, HS + Si + RG, and HS + Si treatments (Table 1). However, exposure to high temperature had resulted in a reduction in shoot biomass ranging from 25 to 40% with the lowest reduction observed in seedlings grown with bacteria and silicates.

Likewise, root biomass significantly increased by 34, 31, and 51% over control (AC) in AC + RG, AC + Si, and AC + Si + RG treatments, respectively, under ambient conditions. Root biomass increment (over control) in seedlings after heat stress was 32, 44 and 62%, respectively, in the presence of bacteria (HS + RG), insoluble silicates (HS + Si) and in the treatment with both silicon and *Rhizobium* sp. IIRR N1 plus *G. diazotrophicus* (HS + Si + RG). Root biomass was lower by 5–23% after the seedlings were subjected to heat stress (Table 1).

Mirroring the trends in seedling biomass, the level of increase in silica content of shoot and root over control with AC + RG, AC + Si and AC + Si + RG was 4, 10 and 19% and 6, 14, and 24%, respectively, when seedlings were grown in ambient temperature. After heat stress, seedlings subjected to HS + RG, HS + Si and HS + Si + RG treatments also had higher silica content than control (HS), with the corresponding increase in shoot and root being 3, 26, 36 and 13%, 28, 44%. Imposition of heat stress decreased silica content in the shoot and root by 10 and 19% (Table 1).

### Physiological characteristics of rice seedlings

Differences in chlorophyll content, electrolyte leakage and relative water content observed between control and treated seedlings and between the two temperature regimes are shown in Table 2. Leaf chlorophyll content increased substantially due to bacterial and silicate treatments, with AC + RG, AC + Si and AC + Si + RG treatments having 30, 13, and 33% higher chlorophyll concentrations respectively, than that of control (AC). Regarding the chlorophyll content of heat-stressed plants, the treatment with silicon and *Rhizobium* sp. IIRR N1 plus *G. diazotrophicus* (HS + Si + RG) recorded a 79% increase in chlorophyll content relative to control (HS) superior to the 37 and 36% increase observed with HS + RG and HS + Si treatments, respectively (Table 2). Following heat stress, 2–29% reduction in chlorophyll content was observed across all treatments compared to ambient temperature.

**Table 2:** Seedling physiological characteristics under ambient and heat stress conditions.

Treatments	Chlorophyll content (mg/g leaf fresh wt)	Relative water content (%)	Electrolyte leakage (%)
Ambient (28 ± 2°C)			
AC	1.71	86.4	47.5
AC + RG	1.97	95.8	33.1
AC + Si	1.80	90.8	41.3
AC + Si + RG	2.22	98.1	31.9
Mean	1.92	92.8	38.4
CD (*P* < 0.05)	0.04	12.7	3.3
CV (%)	9.1	16.7	10.4
Heat stress (45 ± 2°C)			
HS	1.21	71.2	61.1
HS + RG	1.67	89.9	42.6
HS + Si	1.65	82.3	52.3
HS + Si + RG	2.17	92.9	38.7
Mean	1.68	84.1	48.6
CD (*P* < 0.05)	0.01	7.5	4.4
CV (%)	7.3	11.0	11.4

AC, Seedlings grown in the absence of silicates and bacteria under ambient temperature.

AC + RG, Seedlings co-cultured with *Rhizobium* sp. IIRR N1 plus *G. diazotrophicus* bacteria under ambient temperature.

AC + Si, Seedlings co-cultured with solely with insoluble diatomaceous earth and straw under ambient temperature.

AC + Si + RG, Seedlings co-cultured with both silicon and *Rhizobium* sp. IIRR N1 plus *G. diazotrophicus* under ambient temperature.

HS, Seedlings grown in the absence of silicates and bacteria under heat stress condition.

HS + RG, Seedlings co-cultured with *Rhizobium* sp. IIRR N1 plus *G. diazotrophicus* bacteria under heat stress.

HS + Si, Seedlings co-cultured with solely with insoluble diatomaceous earth and straw under heat stress.

HS + Si + RG, Seedlings co-cultured with both silicon and *Rhizobium* sp. IIRR N1 plus *G. diazotrophicus* under heat stress.

Relative water content (RWC) trends showed a similar pattern as that of chlorophyll; higher RWC in leaves of seedlings where treatments have been administered, compared to control in both the temperature regimes (Table 2). High temperature has resulted in water loss from exposed seedlings, with bacterial and silicate-treated seedlings recording a minimum per cent reduction in RWC (5–9%) compared to seedlings under ambient temperature.

Seedlings inoculated with bacteria, treated with insoluble silicates, and the combination of both bacteria and silicates caused a reduction in total electrolyte leakage from seedlings by 30, 13 and 33% and 30, 14 and 37% relative to the respective ambient (AC) and heat-stressed (HS) controls (Table 2). Membrane injury in seedlings due to heat stress was witnessed, and the higher electrolyte leakage values in heat stressed seedlings corroborated the same.

### Antioxidant enzyme activities in rice seedlings

The different treatments comprising silicates and bacteria markedly enhanced the rice seedling antioxidant enzyme activity compared to the control under ambient and heat stress (Table 3). The activities of superoxide dismutase, catalase, ascorbate peroxidase and glutathione reductase were significantly and maximally increased by the combined application of silicates and bacteria (Si + RG) by 25, 38, 45, and 30% under ambient conditions and by 23, 42, 28, and 36% in heat-stressed plants, relative to their corresponding controls. A lower magnitude of increase in the enzymatic antioxidant activities was also observed due to sole treatments with either silicates or bacteria. Heat stress considerably decreased the SOD, CAT, APX and GR activities in seedlings by 3.6–7.1%, 17.1–24.3%, 7.7–19.1%, and 9.4–14.6%, respectively in comparison to non-stressed seedlings subjected to similar conditions (Table 3).

**Table 3:** Seedling antioxidant activities under ambient and heat stress conditions.

Treatments	Superoxide dismutase (Units SOD/mg protein)	Catalase (mM H_2_O_2_/min/ mg protein)	Ascorbate peroxidase (mM ascorbate /min/mg protein)	Glutathione reductase (mM TNB/min/mg protein)
Ambient (28 ± 2°C)				
AC	20.3	8.60	10.46	15.1
AC + RG	23.6	10.56	13.32	18.1
AC + Si	22.3	9.71	12.06	16.8
AC + Si + RG	25.3	11.87	15.13	19.7
Mean	22.9	10.18	12.74	17.4
CD (*P* < 0.05)	0.3	0.2	0.1	0.3
CV (%)	1.6	2.8	1.2	1.0
Heat stress (45/37°C)				
HS	19.1	6.77	9.55	12.9
HS + RG	22.5	8.75	12.05	16.4
HS + Si	21.5	7.35	11.13	14.4
HS + Si + RG	23.5	9.59	12.24	17.5
Mean	21.6	8.11	11.24	15.3
CD (*P* < 0.05)	0.2	0.3	0.1	0.1
CV (%)	1.7	3.4	1.3	2.8

AC, Seedlings grown in the absence of silicates and bacteria under ambient temperature.

AC + RG, Seedlings co-cultured with *Rhizobium* sp. IIRR N1 plus *G. diazotrophicus* bacteria under ambient temperature.

AC + Si, Seedlings co-cultured with solely with insoluble diatomaceous earth and straw under ambient temperature.

AC + Si + RG, Seedlings co-cultured with both silicon and *Rhizobium* sp. IIRR N1 plus *G. diazotrophicus* under ambient temperature.

HS, Seedlings grown in the absence of silicates and bacteria under heat stress condition.

HS + RG, Seedlings co-cultured with *Rhizobium* sp. IIRR N1 plus *G. diazotrophicus* bacteria under heat stress.

HS + Si, Seedlings co-cultured with solely with insoluble diatomaceous earth and straw under heat stress.

HS + Si + RG, Seedlings co-cultured with both silicon and *Rhizobium* sp. IIRR N1 plus *G. diazotrophicus* under heat stress.

### Correlation analysis

**Figure 1:**
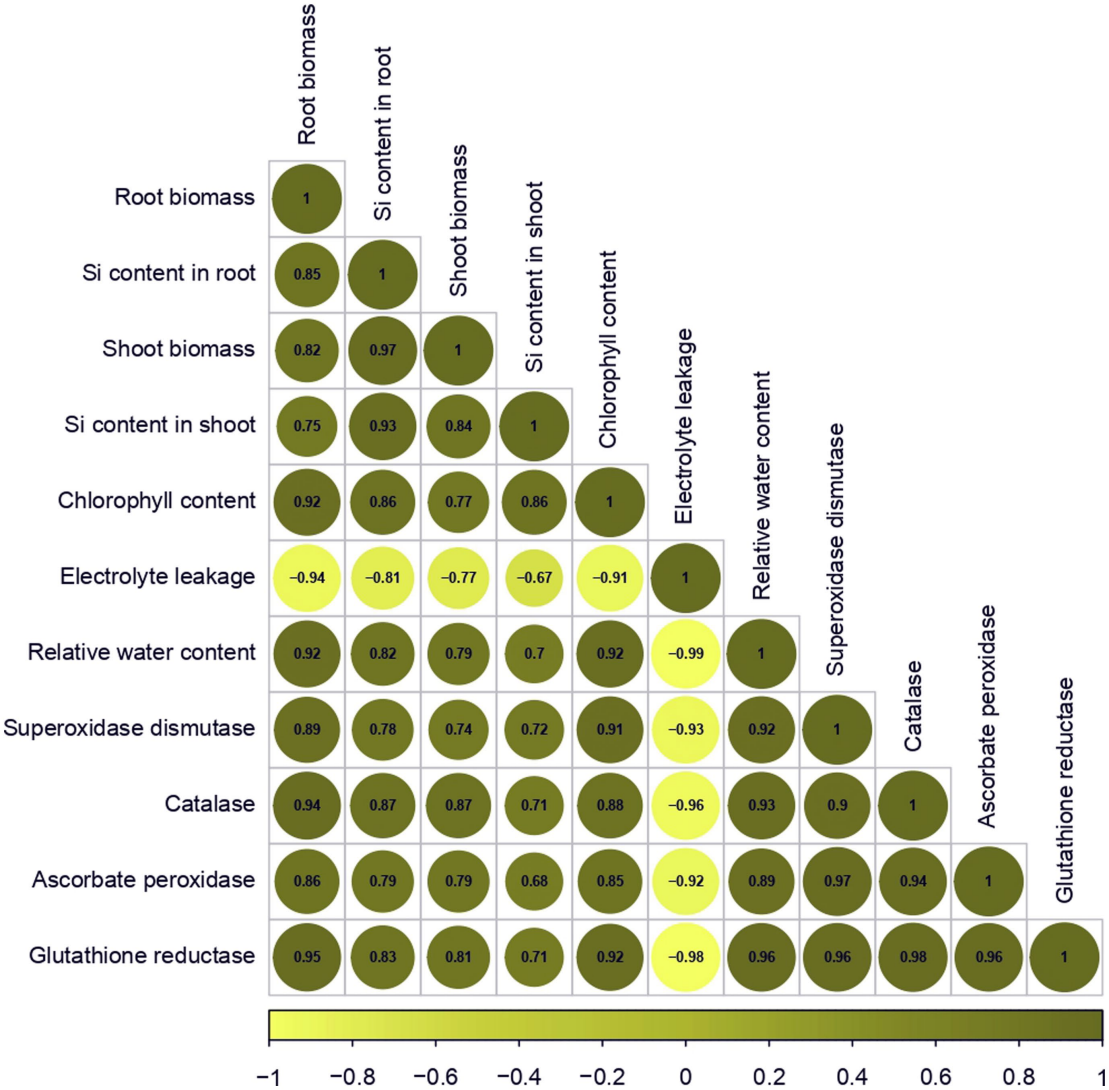
Correlation matrix for morphological, physiological and biochemical variables. Positive and negative correlations are shown as green and yellow circles, respectively. The color legend at the bottom represents the intensities of correlation coefficients.

Correlation analysis revealed a strong association between root and shoot seedling biomass, an indicator of heat stress tolerance, physiological traits, and antioxidant enzymatic activities under ambient and heat stress conditions (Figure 1).

Root biomass showed a significant and positive correlation with Si content in the root (*r* = 0.85), shoot biomass (*r* = 0.82), Si content in the shoot (*r* = 0.75), chlorophyll content (*r* = 0.92), relative water content (*r* = 0.92), superoxide dismutase (*r* = 0.89), catalase (*r* = 0.94), ascorbate peroxidase (*r* = 0.86), glutathione reductase (*r* = 0.95) concentration and a similar positive correlation was also noted with shoot biomass and Si content in both root and shoot (*r* = 0.97 and 0.84), chlorophyll content (*r* = 0.77), relative water content (*r* = 0.79), superoxide dismutase (*r* = 0.74), catalase (*r* = 0.86), ascorbate peroxidase (*r* = 0.79) and glutathione reductase (*r* = 0.81) concentration. In contrast, a significant negative correlation was observed between electrolyte leakage (*r* = −0.94) and root biomass (Figure 1; Supplementary Table S2).

Further, significant positive correlations were also observed among Si content in the root and shoot to chlorophyll content (*r* = 0.86), relative water content (*r* = 0.82 and 0.70), superoxide dismutase (*r* = 0.78 and 0.72), catalase (*r* = 0.87 and 0.70), ascorbate peroxidase (*r* = 0.79 and 0.67) and glutathione reductase (*r* = 0.83 and 0.71) activity while electrolyte leakage (−0.81 and −0.67) was negatively correlated (Figure 1; Supplementary Table S2).

### Heat stress tolerance index

**Figure 2:**
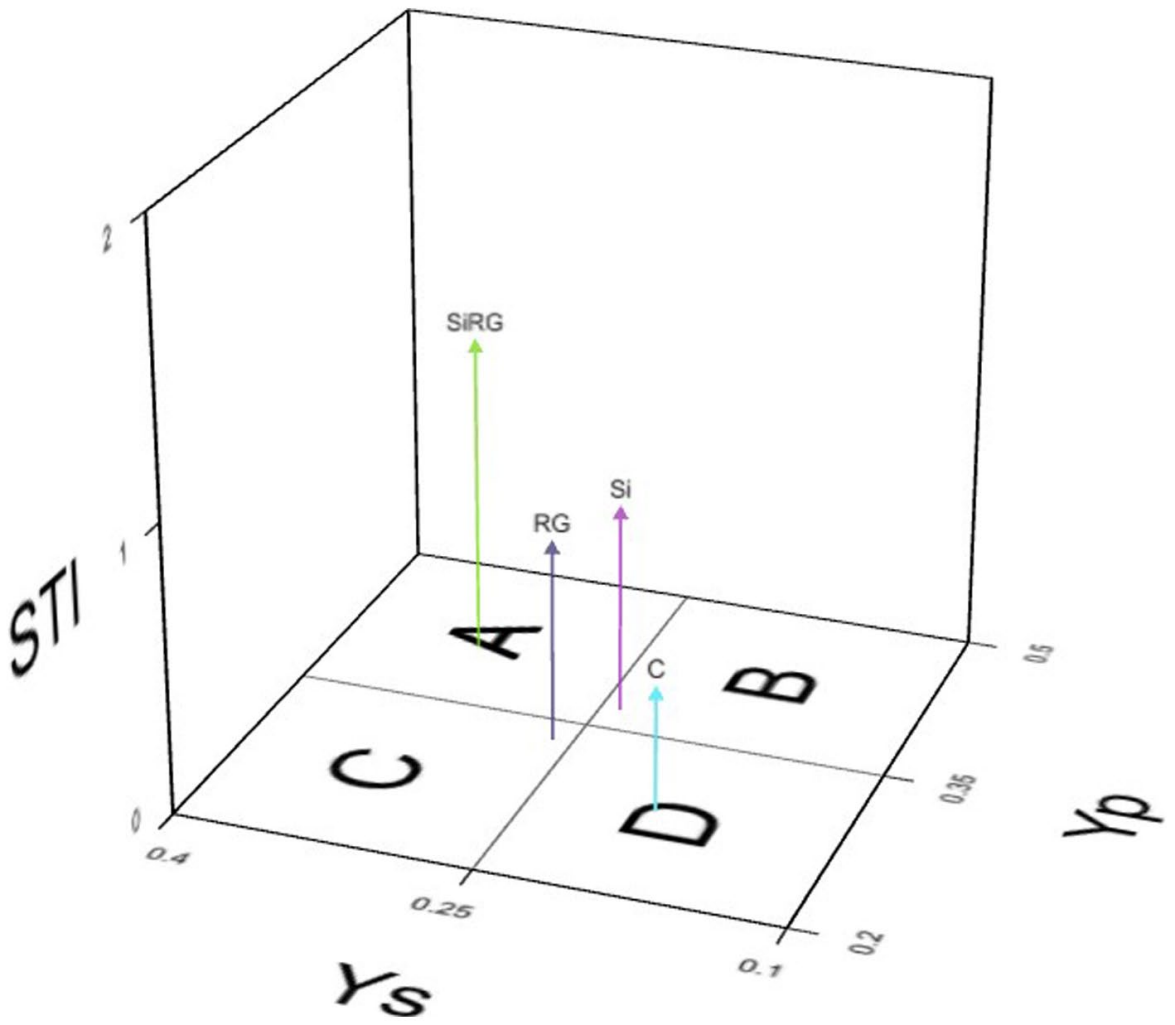
3D plot for the stress tolerance index based on rice seedling biomass under ambient temperature (Yp) and heat stress (Ys).

Heat stress tolerance index (STI) of rice seedlings under normal (Yp) and heat stress (Ys) conditions with different treatments (RG, Si and Si + RG) were calculated using seedling biomass as a screening indicator (Figure 2; Supplementary Table S3) wherein based on Fernandez’s theory, treatments with STI ≥1 were considered stress tolerant. A three-dimensional plot with STI values was computed using iPASTIC, an online toolkit to categorize the treatments into four groups (Figure 2). The treatment with the combined application of both silicates and bacteria was classified as group A, indicating the ability of the treatment to induce uniform performance of rice seedlings under ambient and heat stress conditions. The treatments where the silicates and bacteria were applied individually were categorized into groups B and C, respectively, indicating that treatment with diatomaceous earth and straw could support better seedling growth only under ambient conditions, while inoculation with the two bacteria led to better performance of the seedlings under heat stress conditions (Figure 2).

### Gene expression profiling of HSPs, hormone-related, and silicon transporter genes

**Figure 3:**
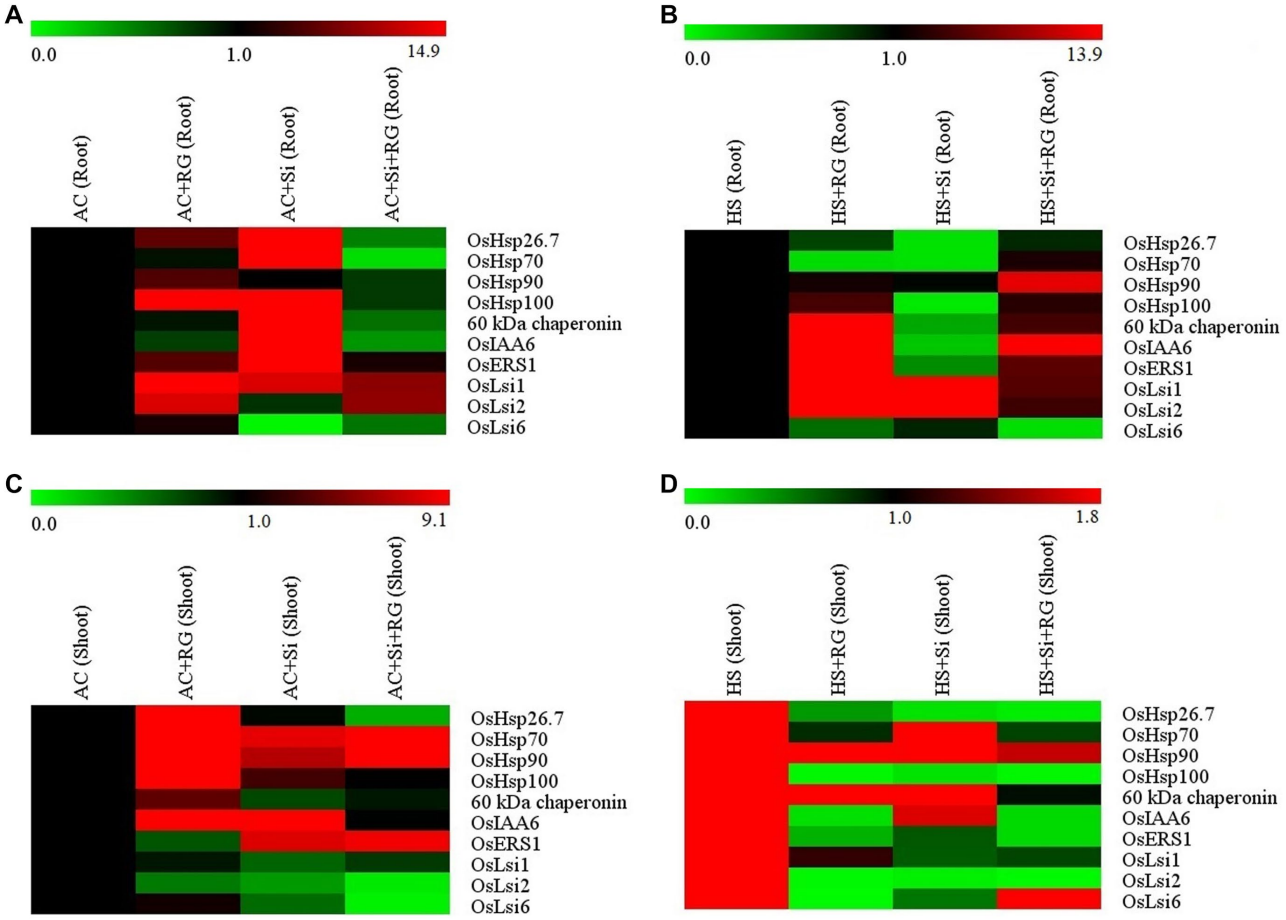
Heatmap representation of HSPs, hormone-related and silicon transporter genes expression profile in root tissue **(A,B)** and shoot tissue **(C,D)** of rice seedlings under ambient and heat stress conditions. The color scale at the top shows relative expression level values. Red indicates a higher expression level, and green indicates a lower expression level.

The expression of selected 10 genes related to HSPs (*viz. OsHsp26.7, OsHsp70, OsHsp90, OsHsp100* and *60 kDa chaperonin*), growth hormones (*OsIAA6, OsER1*) and silicon transporters (*viz. OsLsi1, OsLsi2* and *OsLsi6*) was studied through qRT-PCR of the shoot and root tissues at seedling stage under ambient and heat stress conditions (Figure 3).

Growth of seedlings in the presence of silicates (AC + Si) increased the relative expression of *OsHsp26.7, OsHsp70, OsHsp100* and *60 kDa chaperonin* by a fold change of 12.9, 11.6, 14.9 and 6.6, respectively, in root tissue under ambient conditions (Figure 3A; Supplementary Figure S1). The treatment AC + Si also similarly induced a 5.3 and 3.7-fold change in the expression of the *OsIAA6* and *OsERS1*gene expression, respectively. Inoculation with the bacteria (AC + RG) showed higher expression of silicon transporters genes. *OsLsi1* (1.8-fold change), *OsLsi2* (1.7-fold change) and *OsLsi6* (1.1-fold change) (Figure 3A; Supplementary Figure S1).

In root tissue under heat stress conditions, high relative expression of *OsHsp90* (2.9 and 1.2-fold change), *OsHsp100* (1.3 and 1.6-fold change) and *60 kDa chaperonin* (3.4 and 1.6-fold change) was observed in the treatments HS + RG and HS + Si + RG where the plants were inoculated with bacteria alone or a combination of both silicates and bacteria (Figure 3B; Supplementary Figure S2). Similarly, the transcript levels of both hormone related genes, *OsIAA6* and *OsERS1*, exhibited the highest increase in the treatments HS + RG (5.4 and 4.5-fold change) and HS + Si + RG (4 and 1.8-fold change). The transcript abundance of the two silicon transporter genes, *OsLsi1* and *OsLsi2*, however, were higher in HS + Si treatment (9.8 and 13.9-fold change) followed by HS + RG treatment (3.1 and 3.9-fold change) and HS + Si + RG (1.7 and 1.5-fold change). In contrast, *OsLsi6* transcripts remained at a low level in all the treatments (Figure 3B; Supplementary Figure S2).

In the case of shoot tissue, expression of *OsHsp26.7, OsHsp100, OsHsp70*, and *60 kDa chaperonin* were higher (4.5, 5.9, 5.1, and 1.5-fold change, respectively) with response to AC + RG treatment under ambient conditions (Figure 3C; Supplementary Figure S3) while a 3.7-fold increase in the expression of *OsHsp90* gene was observed when treated with both bacteria and silicates (AC + Si + RG). The hormone-related gene, *OsIAA6*, expression was elevated 9.1 and 2.3-fold with AC + Si and AC + RG treatments, respectively while there was no change in relative expression in the treatment AC + Si + RG. A 2.2 and 2-fold increase was observed in the expression levels of the *OsERS1* gene as influenced by AC + Si + RG and AC + Si treatments, respectively. Among the silicon transporters, transcript levels corresponding to *OsLsi1* and *OsLsi2* did not show much change between the treatments. (Figure 3C; Supplementary Figure S3).

Furthermore, *OsHsp90* was highly expressed under heat stress conditions in shoot tissue treated with HS + Si (1.8-fold change) followed by HS + RG (1.2-fold change; Figure 3D; Supplementary Figure S4). Elevated expression of 60 kDa chaperonin by 1.2-fold (HS + RG) and by 1.1-fold in the HS + Si treatment was observed. In the combined treatment with silicates and bacteria (HS + Si + RG) there was a lower expression of both these genes compared to the control (HS). On the other hand, *OsHsp26.7* and *OsHsp100* expression reduced with the treatments HS + RG followed HS + Si, HS + Si + RG in comparison with heat-stressed control HS (control). However, among the silicon transporters genes, *OsLsi6* showed higher expression (1.6-fold change) in response to HS + Si + RG and showed lower expression with HS + Si and HS + RG. The expression levels of *OsIAA6* and *OsERS1* were lower in all treatments relative to HC (control; Figure 3D; Supplementary Figure S4).

Overall, the expression profiling of *HSPs* (*OsHsp90, OsHsp100* and *60 kDa chaperonin*), hormone-related (*OsIAA6*) and silicon transporters (*OsLsi1* and *OsLsi2*) exhibited higher expression in both shoot and root tissue HS + RG, HS + Si and HS + Si + RG. Thus, comparatively based on HSPs, hormone-related and silicon transporters genes expression profiling shows that the treatment HS + Si + RG (both silicon and *Rhizobium* sp. IIRR N1, *G. diazotrophicus*) augmenting silicon uptake affects the alleviating or coping maximum heat stress conditions compared to bacteria alone (*Rhizobium* sp. IIRR N1 and *G. diazotrophicus*) and silicon alone in rice.

## Discussion

Heat stress severely affects crop production, resulting in global food insecurity (Zhou et al., 2017; Raza, 2020) as it reduces crop yields (Szymanska et al., 2017; Thakur et al., 2021). Among the various abiotic stresses, heat stress causes the most devastating effect on the physiological processes of plant growth, development, and metabolism in several ways (Szymanska et al., 2017; Thakur et al., 2021). Silicon fertilization is emerging as an important agronomic practice to combat the negative effects of biotic and abiotic stresses on plants. However, due to the high cost of synthetically manufactured silica fertilizers (Raza et al., 2023) attention has now shifted to the use of cost-effective and sustainable biological sources of Si like diatomaceous earth (Duboc et al., 2019) and crop residues (Hughes et al., 2020). The insoluble Si present in these sources can be released as soluble phyto-available Si by co-inoculation with silicate solubilizing and decomposer microorganisms. Similarly, plant growth-promoting bacteria colonizing the rhizospheric and endospheric niches and possessing phytohormone modulating capabilities like the production of auxins like IAA and enzymes like 1-aminocyclopropane-1-carboxylate-deaminase (ACC deaminase) can improve the plants’ ability to cope with environmental stresses (Fahad et al., 2015; Souza et al., 2015; Cura et al., 2017; Etesami and Jeong, 2018; Tapera et al., 2018) by reducing the production of stress-induced ethylene.

Our investigation demonstrates that the exogenous application of Si as a mixture of diatomaceous earth and rice straw in combination with silicate solubilizing bacteria, *Rhizobium* sp. IIRR N1 and plant growth-promoting endophytic bacteria *G. diazotrophicus* reduced the adverse effect of heat stress in rice plants by improving the morphological traits that include root and shoot biomass. The results presented in this study concur with earlier reports on exogenous Si application alleviating the adverse effects on plant growth under different abiotic stress conditions including salt stress in rice (Mahdieh et al., 2015), metal toxicity in rice (Chen et al., 2019), and salt stress in okra (Abbas et al., 2017). Our findings were similar to the previous reports on the exogenous application of silicon improving shoot length and biomass production in rice (Agarie et al., 1998), cucumber (Liu et al., 2009), sword fern (Sivanesan et al., 2014), and tomato (Khan et al., 2020) while enhancing the heat stress tolerance. Furthermore, the combined use of Si and PGPRs has been established as a sustainable strategy for alleviating crop abiotic stresses (Rizwan et al., 2015; Kaushal and Wani, 2016; Mahmood et al., 2016). Heat, heavy metal toxicity, nutrient deficiency, salinity, and water deficit are some of the stresses overcome by plants (Verma et al., 2019) due to the interaction of both PGPRs and Si which could be the synergistic result of both available Si and PGPR mediated regulation of plant hormones biosynthesis, i.e., auxins, abscisic acid (ABA), cytokinins, gibberellins, and ethylene (Kim et al., 2014; Yin et al., 2016; Tsukanova et al., 2017). Most of the studies related to the combined application of silicates and plant growth-promoting bacteria involved the use of soluble silicates (Al-Garni et al., 2019; Kubi et al., 2021; Mahmood et al., 2022) unlike the present study wherein inoculation with silicate solubilizing bacteria (*Rhizobium* sp. IIRR N1) could have made available soluble Si from insoluble biogenic silicates as was reported earlier by Chandrakala et al. (2019a). The accelerated release of soluble Si from diatomaceous earth (diatom frustules of amorphous Si), which is highly recalcitrant to solubilization, through bio-dissolution by microbes has been reported earlier (Bidle et al., 2002; Roubeix et al., 2008; Holstein and Hensen, 2010). Microorganisms also play a role in decomposing phytolith silica contained in crop residues such as straw (Alfredsson et al., 2016; de Tombeur et al., 2021).

The effect of inoculation with *Rhizobium* sp. IIRR N1 and *G. diazotrophicus* application were similar to earlier reports on PGPRs mitigating heat stress. Inoculation with *Bacillus cereus*, *Pseudomonas* spp., *Serratia liquefaciens*, *P. fluorescens*, *Pseudomonas putida*, and *Bacillus tequilensis* SSB07 has alleviated heat stress in several crops like tomato, pigeon pea wheat and soybean (Kang et al., 2019). The capability of 1-aminocyclopropane-1-carboxylate (ACC) deaminase activity in bacteria is presently one of the main selection criteria while identifying bacteria for plant stress alleviation (Gupta and Pandey, 2019; Etesami et al., 2020; Orozco-Mosqueda et al., 2023). The enzyme ACC deaminase degrades ACC, the precursor for ethylene in plants into ammonia and alpha-ketobutyrate (nitrogen and carbon nutrient source for the bacteria) resulting in reduced stress ethylene production by plants thereby enabling plants to escape the adverse effect of the gaseous hormone. ACC deaminase producing *Methylobacterium oryzae* (Roy et al., 2022) and *Glutamibacter* sp. (Ji et al., 2020) and *Burkholderia* sp. (Sarkar et al., 2018) inoculation has been reported to induce salt stress tolerance, while *Bradyrhizobium* SUTN9-2 (Sarapat et al., 2020) enhanced rice growth under drought stress condition in rice. Submergence tolerance also have been induced in rice treated with ACC deaminase producing *Microbacterium* sp. AR-ACC2, *Paenibacillus* sp. ANR-ACC3, and *Methylophaga* sp. AR-ACC3 which resulted in significant growth improvement growth parameters when compared with non-treated seeds (Bal and Adhya, 2021). There is a paucity of studies on the interactions between rice and bacteria and their role in inducing heat stress tolerance. Both bacteria used in this study possess unique traits that could have played a role in improving rice seedlings’ tolerance to heat stress. *Rhizobium* sp. IIRR N1, in addition to silicate solubilization potential, can produce IAA and ACC deaminase, which could have modulated stress ethylene levels in seedlings while inducing heat stress tolerance (Chandrakala et al., 2019b). *G. diazotrophicus* is well known as a drought stress alleviator (Filgueiras et al., 2020; Silva et al., 2020), though its effect on plants during heat stress has not been studied.

The highest Si content in root and shoot in this study were observed to be in treatment with silicon and *Rhizobium* sp. IIRR N1 plus *G. diazotrophicus* (H + Si + RG) followed by those treated with silicon alone and treatment of *Rhizobium* sp. IIRR N1 plus *G. diazotrophicus* under heat stress conditions. Diatomaceous earth (Marxen et al., 2016) and rice straw incorporation (Pati et al., 2016; Sandhya and Prakash, 2019) in soil have been reported to influence the uptake of Si by rice plants. However, both diatomaceous earth and rice straw show variability in their ability to supply Si to plants (Duboc et al., 2019; Zellener et al., 2021) due to slow and inconsistent release of soluble Si that does not match to the plant demands. The inoculation of these Si source with silicate solubilizing bacteria is expected to improve the efficiency of these silicate sources. Thus, our findings on morphological traits demonstrate that exogenous application of both silicon and *Rhizobium* sp. IIRR N1 plus *G. diazotrophicus* (H + Si + RG) can alleviate the effect of heat stress in rice seedlings plants enhancing plant growth attributes, such as root length, shoot length, silicon content in root and shoot and seedling biomass production.

The combined application of silicon and *Rhizobium* sp. IIRR N1 plus *G. diazotrophicus* (H + Si + RG) significantly increased chlorophyll content, relative water content in leaf followed by *Rhizobium* sp. IIRR N1 plus *G. diazotrophicus* (HS + RG) and silicon alone (HS + Si) respectively under heat stress conditions. Our findings on the chlorophyll content and relative water content concur with earlier studies in rice crops (Ma et al., 1989; Tamai and Ma, 2008; Detmann et al., 2012), which demonstrated that Si application increased plant growth, chlorophyll content, photosynthetic activity and subsequently enhanced productivity. In our results, electrolyte leakage was lowest in the combined application of silicon and *Rhizobium* sp. IIRR-1 plus *G. diazotrophicus* followed by *Rhizobium* sp. IIRR-1 plus *G. diazotrophicus* and silicon alone, respectively, under heat stress conditions. It has been reported that heat stress causes electrolyte leakage in higher plants grown without Si or under minimal Si applied conditions (Agarie et al., 1998). In rice, increasing the level of Si reduces the electrolyte leakage from leaf tissues, indicating that the concentration of Si in leaf tissue is correlated with cell wall polysaccharides level and also indicates Si role in maintaining thermal stability of lipids in cell membranes (Agarie et al., 1998).

Heat stress induces the production of highly toxic reactive oxygen species (ROS) leading to oxidative stress (Sarkar et al., 2018), and damages the various cellular, sub-cellular membranes, and macro-molecules causing disturbances in cellular homeostasis (Foyer and Noctor, 2011). Also, ROS molecules disturb the photosynthetic system (PSI and PSII) and cell membrane stability by triggering electrolyte leakage and autocatalytic lipid peroxidation (Tiwari and Yadav, 2019). Plants cope with oxidative damage by enhancing the endogenous content of catalase, peroxidase, superoxide dismutase, glutathione reductase, guaiacol peroxidase, ascorbate peroxidase, and dehydroascorbate reductase which are enzymatic antioxidants that protects against the oxidative stress produced during to abiotic stresses (Etesami and Jeong, 2018; Verma et al., 2019). It has been reported that PGPR-induced antioxidant enzymes production has the potential to mitigate the abiotic stresses in plants by reducing ROS (Etesami and Jeong, 2018). Also, exogenous application of Si may alleviate oxidative damage during abiotic stresses in plants by modifying the antioxidant machinery consisting of both enzymatic and non-enzymatic components (Zhu and Gong, 2014; Kim et al., 2017).

Our findings revealed that both silicon and *Rhizobium* sp. IIRR N1 plus *G. diazotrophicus* application under heat-stressed rice seedlings increased the enzymatic antioxidants activities in line with earlier reports on tomato soybean and wheat crop (Ali et al., 2009, 2011; Meena et al., 2015; Choudhary et al., 2016; Bisht et al., 2020). In wheat, *Pseudomonas putida AKMP7* has enhanced the shoot and root length, dry biomass, tillers number, grain formation, and decreased membrane injury and antioxidant enzyme activities such as SOD, APX, and CAT under heat stress conditions (Ali et al., 2011). In wheat, auxin-producing *Azospirillum brasilense* was shown to alleviate heat stress by maintaining water status (Choudhary et al., 2016). In rice, combined application of bacterial inoculants and nano-silicon improved antioxidant activities under salt stress conditions (Alharbi et al., 2022). Kim et al. (2017) also reported that combined exogenous application of PGPRs and silicon may alleviate oxidative damage in crops by inducing ROS, CAT, POD, SOD, GR and APOD enzymatic activities under various abiotic stress conditions.

At the molecular level, the HSP genes *viz. OsHsp90, OsHsp100, 60 kDa chaperonin,* two phyto-hormone genes *viz*. *OsIAA6* and *OsERS1* and two silicon transporter genes, *OsLsi1* and *OsLsi2* showed higher expression in the treatment of HS + RG and HS + Si + RG over control (HS) in root tissues under heat stress conditions. Whereas, in shoot tissues, two HSP genes *viz*. *OsHsp90*, *60 kDa chaperonin,* and *OsIAA6* showed higher expression in HS + Si and HS + RG. The *OsLsi6* silicon transporter gene showed higher expression in response to HS + Si + RG treatment in shoot tissue under heat-stress conditions. It has been reported that plants overcome heat stress by mechanistic response via activation of conserved pathways, including overexpression of ABA-responsive genes, calcium-sensing proteins, HSPs-induced protein folding, and ROS-scavenging genes (Janni et al., 2020). Plants develop a diverse array of heat stress tolerance mechanisms including biosynthesis of heat shock proteins (HSPs), biosynthesis of specific phytohormones, and scavenge the ROS mechanism (Khan et al., 2020; Raza et al., 2021; Haider et al., 2021a,b). Our findings while profiling the gene expression of HSPs, hormone-related, and silicon transporters genes are concurrent with previous results on the combined application of PGPRs and Si that have documented enhanced expression of these genes while exhibiting tolerance to heat stress (Tiwari et al., 2016; Janni et al., 2020; Khan et al., 2020). It has been reported that Si-based activation of HSFs and HSPs interact with various signaling cascades triggered by Ca^2+,^ phospholipids, phytohormones, and H_2_O_2_, to reduce the effects of heat stress in crop plants (Liu et al., 2015; Sharma et al., 2019). The role of Si in inducing heat stress tolerance by stimulating heat shock proteins, antioxidant system, and phytohormone production in tomatoes was demonstrated by Khan et al. (2020).

In rice, silicon transporters *viz*. *OsLsi1* and *OsLsi2* are present in the plasma membrane of rice plant cells and are located on the distal side of the cell and proximal side of the cell and play a role in Si influx and efflux, respectively, (Ma et al., 2007). In rice, Si is taken up by rice roots in the available form of silicic acid (SiOH4) through Si influx (*Lsi1*) and efflux transporters (*Lsi2*) genes and then translocated into aerial organs, i.e., shoots through xylem transpiration stream unloaded by *Lsi6* (Yamaji et al., 2008, 2015). Tiwari et al. (2016) demonstrated that they induce the expression of stress-responsive genes of rice seedlings in response to *B. amyloliquefaciens* NBRI-SN12 and enhanced the accumulation of osmoprotectants mitigate the heat-stress conditions. Similarly, *Pseudomonas* sp. AKM-P6 treated sorghum seedlings showed higher expression and accumulations of HSPs and generating EPSs, which leads to tolerance to the heat stress conditions (Sandhya et al., 2009).

Thus, our findings revealed exogenous application of both silicon and *Rhizobium* sp. IIRR N1 plus *G. diazotrophicus* (H + Si + RG) showed higher expression of silicon transporters genes *OsLsi1, OsLsi2,* and *OsLsi6* in root and shoot tissue under heat stress conditions, suggesting enhanced Si uptake and played a role in mitigating heat stress. This role might be due to the uptake of Si from root to shoot and Si in the shoot provides strength to leaf and shoot tissue by minimizing the adverse effects leading to alleviating the heat stress in rice seedlings. This role might be attributed to the accumulation of Si in the shoots, providing additional strength to the leaf and stem structure to minimize the adverse effects of heat and drought stress, hence leading to a higher tolerance.

## Conclusion

Globally, reduction in yield of crops due to heat stress is a major concern. Silicon dependent stress emancipation of plants is gaining popularity as a reliable, eco-friendly strategy to meet global food demands in a sustainable manner under stress agriculture. The results obtained in this study demonstrates that mixed bacterial inoculum of *Rhizobium* sp. IIRR N1 and *Gluconacetobacter diazotrophicus* along with insoluble silicates like diatomaceous earth and rice straw when used in combination as a organo-mineral biofertilizer, can result in conferring heat stress tolerance to a heat susceptible rice genotype.

## Data availability statement

The original contributions presented in the study are publicly available. The datasets generated for this study can be found at the link: https://www.ncbi.nlm.nih.gov/nuccore/KY348774.1 in the NCBI GenBank, Accession number KY34877.

## Author contributions

LC and CC: conceptualization and methodology. LC, CC, and AP: data curation, formal analysis, software, and validation. LC, AP, CC, and BS: investigation and visualization. LC, CC AP, RG, and VK: resources. LC, RS, CC, and BS: supervision. RS: writing—original draft. LC, CC, AP BS, VK, RG, and RS: writing review and editing. All authors contributed to the article and approved the submitted version.

## Conflict of interest

The authors declare that the research was conducted in the absence of any commercial or financial relationships that could be construed as a potential conflict of interest.

## Publisher’s note

All claims expressed in this article are solely those of the authors and do not necessarily represent those of their affiliated organizations, or those of the publisher, the editors and the reviewers. Any product that may be evaluated in this article, or claim that may be made by its manufacturer, is not guaranteed or endorsed by the publisher.

## Footnotes

^1^http://quantprime.mpimp-golm.mpg.de/

## References

[ref1] Abbas T. Sattar A. Ijaz M. Aatif M. Khalid S. Sher A. (2017). Exogenous silicon application alleviates salt stress in okra. Hortic. Environ. Biotechnol. 58, 342–349. doi: 10.1007/s13580-017-0247-5

[ref2] Adhikari A. Khan M. A. Lee K. E. Kang S. M. Dhungana S. K. Bhusal N.. (2020). The halotolerant rhizobacterium-*pseudomonas koreensis* mu2 enhances inorganic silicon and phosphorus use efficiency and augments salt stress tolerance in soybean (*Glycine max* L.). Microorganisms 8:1256. doi: 10.3390/microorganisms8091256, PMID: 32825007 PMC7570339

[ref3] Aebi H. Lester P. (1984). Catalase in vitro. Methods Enzymol 105, 121–126. doi: 10.1016/S0076-6879(84)05016-3 6727660

[ref4] Agarie S. Hanaoka N. Ueno O. Miyazaki A. Kubota F. Agata W.. (1998). Effects of silicon on tolerance to water deficit and heat stress in rice plants (*Oryza sativa* L.), monitored by electrolyte leakage. Plant. Prod. Sci. 1, 96–103. doi: 10.1626/pps.1.96

[ref5] Aghamolki M. T. K. Yusop M. K. Oad F. C. Zakikhani H. Jaafar H. Z. Kharidah S.. (2014). Heat stress effects on yield parameters of selected rice cultivars at reproductive growth stages. J. Food Agric. Environ. 12, 741–746.

[ref6] Alfredsson H. Clymans W. Stadmark J. Conley D. Rousk J. (2016). Bacterial and fungal colonization and decomposition of submerged plant litter: consequences for biogenic silica dissolution. FEMS Microbiol. Ecol. 92:fiw011. doi: 10.1093/femsec/fiw011, PMID: 26790464 PMC4749722

[ref7] Al-Garni S. M. S. Khan M. M. A. Bahieldin A. (2019). Plant growth-promoting bacteria and silicon fertilizer enhance plant growth and salinity tolerance in *Coriandrum sativum*. J. Plant Interact. 14, 386–396. doi: 10.1080/17429145.2019.1641635

[ref8] Alharbi K. Osman H. S. Rashwan E. Hafez E. M. Omara A. E. D. (2022). Stimulating the growth, anabolism, antioxidants, and yield of rice plants grown under salt stress by combined application of bacterial inoculants and nano-silicon. Plants 11:3431. doi: 10.3390/plants11243431, PMID: 36559542 PMC9787420

[ref9] Ali M. G. Ahmed M. Ibrahim M. M. El Baroudy A. A. Ali E. F. Shokr M. S.. (2022). Optimizing sowing window, cultivar choice, and plant density to boost maize yield under RCP8. 5 climate scenario of CMIP5. Int. J. Biometeorol. 66, 971–985. doi: 10.1007/s00484-022-02253-x, PMID: 35149894

[ref10] Ali S. Z. Sandhya V. Grover M. Kishore N. Rao L. V. Venkateswarlu B. (2009). *Pseudomonas* sp. strain AKM-P6 enhances tolerance of sorghum seedlings to elevated temperatures. Biol. Fertil. Soils 46, 45–55. doi: 10.1007/s00374-009-0404-9

[ref11] Ali S. Z. Sandhya V. Grover M. Linga V. R. Bandi V. (2011). Effect of inoculation with a thermotolerant plant growth promoting *Pseudomonas putida* strain AKMP7 on growth of wheat (*Triticum* spp.) under heat stress. J. Plant Interact. 6, 239–246. doi: 10.1080/17429145.2010.545147

[ref12] Arnon D. I. (1949). Copper enzyme in isolated chloroplasts: polyphenol oxidase in *Beta vulgaris*. Plant Physiol. 24, 1–15. doi: 10.1104/pp.24.1.1, PMID: 16654194 PMC437905

[ref13] Asghari B. Khademian R. Sedaghati B. (2020). Plant growth promoting rhizobacteria (PGPR) confer drought resistance and stimulate biosynthesis of secondary metabolites in pennyroyal (*Mentha pulegium* L.) under water shortage condition. Sci. Hortic. 263:109132. doi: 10.1016/j.scienta.2019.109132

[ref14] Bal H. B. Adhya T. K. (2021). Alleviation of submergence stress in rice seedlings by plant growth-promoting rhizobacteria with ACC deaminase activity. Front. Sustain. Food Syst. 5:606158. doi: 10.3389/fsufs.2021.606158

[ref15] Berahim Z. Omar H. M. Zakaria N. Ismail M. R. Rosle R. Roslin N. A.. (2021). Silicon improves yield performance by enhancement in physiological responses, crop imagery, and leaf and culm sheath morphology in new rice line, Padi U Putra. Biol. Med. Res. Int. 2021:6679787. doi: 10.1155/2021/6679787, PMID: 34159198 PMC8187073

[ref16] Beyer W. F. Fridovich I. (1987). Assaying for superoxide dismutase activity: some large consequence of minor change in conditions. Anal. Biochem. 161, 559–566. doi: 10.1016/0003-2697(87)90489-1, PMID: 3034103

[ref17] Bidle K. D. Manganelli M. Azam F. (2002). Regulation of oceanic silicon and carbon preservation by temperature control on bacteria. Science 298, 1980–1984. doi: 10.1126/science.1076076 12471255

[ref18] Bisht N. Mishra S. K. Chauhan P. S. (2020). *Bacillus amyloliquefaciens* inoculation alters physiology of rice (*Oryza sativa* L. var. IR-36) through modulating carbohydrate metabolism to mitigate stress induced by nutrient starvation. Int. J. Biol. Macromol. 143, 937–951. doi: 10.1016/j.ijbiomac.2019.09.154, PMID: 31739073

[ref19] Chandel G. Dubey M. Meena R. (2013). Differential expression of heat shock proteins and heat stress transcription factor genes in rice exposed to different levels of heat stress. J. Plant Biochem. Biotechnol. 22, 277–285. doi: 10.1007/s13562-012-0156-8

[ref20] Chandrakala C. Voleti S. R. Bandeppa S. Kumar N. S. Latha P. C. (2019a). Silicate solubilization and plant growth promoting potential of *Rhizobium* sp. isolated from rice rhizosphere. Silicon 11, 2895–2906. doi: 10.1007/s12633-019-0079-2

[ref21] Chandrakala C. Voleti S. R. Bandeppa S. Rajani G. Prasad Babu K. V. Phule A. S.. (2019b). Effect of ACC deaminase-producing bacteria on germination and seedling growth of rice under heat stress. J. Rice Res. 10, 45–51. doi: 10.3389/fmicb.2019.01506

[ref22] Chen D. Chen D. Xue R. Long J. Lin X. Lin Y.. (2019). Effects of boron, silicon and their interactions on cadmium accumulation and toxicity in rice plants. J. Hazard. Mater. 367, 447–455. doi: 10.1016/j.jhazmat.2018.12.111, PMID: 30611037

[ref23] Choudhary D. K. Kasotia A. Jain S. Vaishnav A. Kumari S. Sharma K. P.. (2016). Bacterial-mediated tolerance and resistance to plants under abiotic and biotic stresses. J. Plant Growth Regul. 35, 276–300. doi: 10.1007/s00344-015-9521-x

[ref24] Constantinescu-Aruxandei D. Lupu C. Oancea F. (2020). Siliceous natural nanomaterials as biorationals-plant protectants and plant health strengtheners. Agronomy 10:1791. doi: 10.3390/agronomy10111791

[ref25] Cooke J. Leishman M. R. (2011). Is plant ecology more siliceous than we realise? Trends Plant Sci. 16, 61–68. doi: 10.1016/j.tplants.2010.10.003, PMID: 21087891

[ref26] Cura J. A. Franz D. R. Filosofía J. E. Balestrasse K. B. Burgueño L. E. (2017). Inoculation with *Azospirillum* sp. and *Herbaspirillum* sp. bacteria increases the tolerance of maize to drought stress. Microorganisms 5:41. doi: 10.3390/microorganisms5030041, PMID: 28933739 PMC5620632

[ref27] Dastogeer K. M. Zahan M. I. Rhaman M. S. Sarker M. S. Chakraborty A. (2022). Microbe-mediated thermotolerance in plants and pertinent mechanisms-a meta-analysis and review. Front. Microbiol. 13:833566. doi: 10.3389/fmicb.2022.833566, PMID: 35330772 PMC8940538

[ref28] de Tombeur F. Roux P. Cornelis J. T. (2021). Silicon dynamics through the lens of soil-plant-animal interactions: perspectives for agricultural practices. Plant Soil 467, 1–28. doi: 10.1007/s11104-021-05076-8

[ref29] Debona D. Rodrigues F. A. Datnoff L. E. (2017). Silicon’s role in abiotic and biotic plant stresses. Annu. Rev. Phytopathol. 55, 85–107. doi: 10.1146/annurev-phyto-080516-035312 28504920

[ref30] Detmann K. C. Araújo W. L. Martins S. C. Sanglard L. M. V. P. Reis J. V. Detmann E.. (2012). Silicon nutrition increases grain yield, which, in turn, exerts a feed-forward stimulation of photosynthetic rates via enhanced mesophyll conductance and alters primary metabolism in rice. New Phytol. 196, 752–762. doi: 10.1111/j.1469-8137.2012.04299.x 22994889

[ref31] Devireddy A. R. Tschaplinski T. J. Tuskan G. A. Muchero W. Chen J. G. (2021). Role of reactive oxygen species and hormones in plant responses to temperature changes. Int. J. Mol. Sci. 22:8843. doi: 10.3390/ijms22168843, PMID: 34445546 PMC8396215

[ref32] Ding Z. Ali E. F. Elmahdy A. M. Ragab K. E. Seleiman M. F. Kheir A. M. (2021). Modeling the combined impacts of deficit irrigation, rising temperature and compost application on wheat yield and water productivity. Agric. Water Manag. 244:106626. doi: 10.1016/j.agwat.2020.106626

[ref33] Duboc O. Robbe A. Santner J. Folegnani G. Gallais P. Lecanuet C.. (2019). Silicon availability from chemically diverse fertilizers and secondary raw materials. Environ. Sci. Technol. 53, 5359–5368. doi: 10.1021/acs.est.8b06597, PMID: 30994336

[ref34] Ejaz M. Abbas G. Fatima Z. Iqbal P. Raza M. A. Kheir A.. (2022). Modelling climate uncertainty and adaptations for soybean-based cropping system. Int. J. Plant Prod. 16, 235–250. doi: 10.1007/s42106-022-00190-8

[ref35] Elavarthi S. Martin B. (2010). Spectrophotometric assays for antioxidant enzymes in plants. Methods Mol. Biol. 639, 273–280. doi: 10.1007/978-1-60761-702-0_16 20387052

[ref360] Elliott C.L. Snyder G. H. (1991). Autoclave-induced digestion for the colorimetric determination of silicon in rice straw. J Agric Food Chem 39, 1118–1119. doi: 10.1021/jf00006a024

[ref36] Epstein E. (1999). Silicon. Annu. Rev. Plant Physiol. Plant Mol. Biol. 50, 641–664. doi: 10.1146/annurev.arplant.50.1.641 15012222

[ref37] Etesami H. Jeong B. R. (2018). Silicon (Si): review and future prospects on the action mechanisms in alleviating biotic and abiotic stresses in plants. Ecotoxicol. Environ. Saf. 147, 881–896. doi: 10.1016/j.ecoenv.2017.09.063, PMID: 28968941

[ref38] Etesami H. Maheshwari D. K. (2018). Use of plant growth promoting rhizobacteria (PGPRs) with multiple plant growth promoting traits in stress agriculture: action mechanisms and future prospects. Ecotoxicol. Environ. Saf. 156, 225–246. doi: 10.1016/j.ecoenv.2018.03.013, PMID: 29554608

[ref39] Etesami H. Noori F. Ebadi A. Reiahi Samani N. (2020). “Alleviation of stress-induced ethylene-mediated negative impact on crop plants by bacterial ACC deaminase: perspectives and applications in stressed agriculture management,” in Plant microbiomes for sustainable agriculture sustainable development and biodiversity. eds. Yadav A. N. Singh J. Rastegari A. A. Yadav N. (Cham: Springer International Publishing), 287–315.

[ref40] Fahad S. Hussain S. Bano A. Saud S. Hassan S. Shan D.. (2015). Potential role of phytohormones and plant growth-promoting rhizobacteria in abiotic stresses: consequences for changing environment. Environ. Sci. Pollut. Res. 22, 4907–4921. doi: 10.1007/s11356-014-3754-2 25369916

[ref41] Farooq M. A. Dietz K. J. (2015). Silicon as versatile player in plant and human biology: overlooked and poorly understood. Front. Plant Sci. 6:994. doi: 10.3389/fpls.2015.00994 26617630 PMC4641902

[ref42] Fernandez G. C. J. (1992). “Effective selection criteria for assessing plant stress tolerance,” in Adaptation of food crops to temperature and water stress. ed. Kuo C. G. (Shanhua, Taiwan: Asian Vegetable Research and Development Center), 257–270.

[ref43] Filgueiras L. Silva R. Almeida I. Vidal M. Baldani J. I. Meneses C. H. (2020). *Gluconacetobacter diazotrophicus* mitigates drought stress in *Oryza sativa* L. Plant Soil 451, 57–73. doi: 10.1007/s11104-019-04163-1

[ref44] Finch-Savage W. E. Bassel G. W. (2016). Seed vigour and crop establishment: extending performance beyond adaptation. J. Exp. Bot. 67, 567–591. doi: 10.1093/jxb/erv490, PMID: 26585226

[ref45] Foyer C. H. Noctor G. (2011). Ascorbate and glutathione: the heart of the redox hub. Plant Physiol. 155, 2–18. doi: 10.1104/pp.110.167569, PMID: 21205630 PMC3075780

[ref46] Frew A. Weston L. A. Reynolds O. L. Gurr G. M. (2018). The role of silicon in plant biology: a paradigm shift in research approach. Ann. Bot. 121, 1265–1273. doi: 10.1093/aob/mcy009, PMID: 29438453 PMC6007437

[ref47] Govindaraj M. Pattanashetti S. K. Patne N. Kanatti A. (2018). Breeding cultivars for heat stress tolerance in staple food crops. Next Gener. Plant Breed. 45. doi: 10.5772/intechopen.76480

[ref48] Gupta S. Pandey S. (2019). Unravelling the biochemistry and genetics of ACC deaminase-an enzyme alleviating the biotic and abiotic stress in plants. Plant Gene 18:100175. doi: 10.1016/j.plgene.2019.100175

[ref49] Haider S. Iqbal J. Naseer S. Yaseen T. Shaukat M. Bibi H.. (2021a). Molecular mechanisms of plant tolerance to heat stress: current landscape and future perspectives. Plant Cell Rep. 40, 2247–2271. doi: 10.1007/s00299-021-02696-3 33890138

[ref50] Haider S. Rehman S. Ahmad Y. Raza A. Tabassum J. Javed T.. (2021b). In silico characterization and expression profiles of heat shock transcription factors (HSFs) in maize (*Zea mays* L.). Agronomy 11:2335. doi: 10.3390/agronomy11112335

[ref51] Hamayun M. Sohn E. Y. Khan S. A. Shinwari Z. K. Khan A. L. Lee I.-J.. (2010). Silicon alleviates the adverse effects of salinity and drought stress on growth and endogenous plant growth hormones of soybean (*Glycine Max* L.). Pakistan J. Bot. 42, 1713–1722.

[ref52] Haneklaus S. Bloem E. Schnug E. (2018). Hungry plants-a short treatise on how to feed crops under stress. Agriculture 8:43. doi: 10.3390/agriculture8030043

[ref53] Hasanuzzaman M. Bhuyan M. H. M. Parvin K. Bhuiyan T. F. Anee T. I. Nahar K.. (2020). Regulation of ROS metabolism in plants under environmental stress: a review of recent experimental evidence. Int. J. Mol. Sci. 21:8695. doi: 10.3390/ijms21228695, PMID: 33218014 PMC7698618

[ref54] Haynes R. J. (2017). Significance and role of Si in crop production. Adv. Agron. 146, 83–166. doi: 10.1016/bs.agron.2017.06.001

[ref55] Heckman J. (2013). Silicon: a beneficial substance. Better Crop. 97, 14–16.

[ref56] Holstein J. M. Hensen C. (2010). Microbial mediation of benthic biogenic silica dissolution. Geo-Mar. Lett. 30, 477–492. doi: 10.1007/s00367-009-0181-3

[ref57] Hughes H. J. Hung D. T. Sauer D. (2020). Silicon recycling through rice residue management does not prevent silicon depletion in paddy rice cultivation. Nutr. Cycl. Agroecosyst. 118, 75–89. doi: 10.1007/s10705-020-10084-8

[ref58] Hussain I. Parveen A. Rasheed R. Ashraf M. A. Ibrahim M. Riaz S.. (2019). Exogenous silicon modulates growth, physio-chemicals and antioxidants in barley (*Hordeum vulgare* L.) exposed to different temperature regimes. Silicon 11, 2753–2762. doi: 10.1007/s12633-019-0067-6

[ref400] Jagadish S. V. K. Way D. A. Sharkey T. D. (2021). Plant heat stress: Concepts directing future research. Plant Cell Environ, 44, 1992–2005. doi: 10.1111/pce.14050, PMID: 33745205

[ref59] Jambunathan N. (2010). Determination and detection of reactive oxygen species (ROS), lipid peroxidation and electrolyte leakage in plants. Methods Mol. Biol. 639, 292–298. doi: 10.1007/978-1-60761-702-0_18, PMID: 20387054

[ref60] Janni M. Gulli M. Maestri E. Marmiroli M. Valliyodan B. Nguyen H. T.. (2020). Molecular and genetic bases of heat stress responses in crop plants and breeding for increased resilience and productivity. J. Exp. Bot. 71, 3780–3802. doi: 10.1093/jxb/eraa034, PMID: 31970395 PMC7316970

[ref61] Ji J. Yuan D. Jin C. Wang G. Li X. Guan C. (2020). Enhancement of growth and salt tolerance of rice seedlings (*Oryza sativa* L.) by regulating ethylene production with a novel halotolerant PGPR strain *Glutamicibacter* sp. YD01 containing ACC deaminase activity. Acta Physiol. Plant. 42, 1–17. doi: 10.1007/s11738-020-3034-3

[ref300] Jinger D. Devi M. T. Dhar S. Dass A. Sharma V. K. Vijayakumar S. (2020). Silicon application mitigates abiotic stresses in rice: A review. Indian J Agric. Sci. 90, 2043–50.

[ref62] Kang S. M. Khan A. L. Waqas M. Asaf S. Lee K. E. Park Y. G.. (2019). Integrated phytohormone production by the plant growth-promoting rhizobacterium *Bacillus tequilensis* SSB07 induced thermotolerance in soybean. J. Plant Interact. 14, 416–423. doi: 10.1080/17429145.2019.1640294

[ref63] Kaushal M. Wani S. P. (2016). Plant-growth-promoting rhizobacteria: drought stress alleviators to ameliorate crop production in drylands. Ann. Microbiol. 66, 35–42. doi: 10.1007/s13213-015-1112-3

[ref64] Khan A. Khan A. L. Imran M. Asaf S. Kim Y. H. Bilal S.. (2020). Silicon-induced thermotolerance in *Solanum lycopersicum* L. via activation of antioxidant system, heat shock proteins, and endogenous phytohormones. BMC Plant Biol. 20:248. doi: 10.1186/s12870-020-02456-7, PMID: 32493420 PMC7268409

[ref65] Kim Y. H. Khan A. L. Hamayun M. Kang S. M. Beom Y. J. Lee I. J. (2011). Influence of short-term silicon application on endogenous physio-hormonal levels of *Oryza sativa* L. under wounding stress. Biol. Trace Elem. Res. 144, 1175–1185. doi: 10.1007/s12011-011-9047-4, PMID: 21465280

[ref66] Kim Y. N. Khan M. A. Kang S. M. Hamayun M. Lee I. J. (2020). Enhancement of drought-stress tolerance of *Brassica oleracea var. italica* L. by newly isolated *Variovorax* sp. YNA59. J. Microbiol. Biotechnol. 30, 1500–1509. doi: 10.4014/jmb.2006.06010, PMID: 32807757 PMC9728237

[ref67] Kim Y. H. Khan A. L. Waqas M. Lee I. J. (2017). Silicon regulates antioxidant activities of crop plants under abiotic-induced oxidative stress: a review. Front. Plant Sci. 8:510. doi: 10.3389/fpls.2017.00510, PMID: 28428797 PMC5382202

[ref68] Kim Y. H. Khan A. L. Waqas M. Shim J. K. Kim D. H. Lee K. Y.. (2014). Silicon application to rice root zone influenced the phytohormonal and antioxidant responses under salinity stress. J. Plant Growth Regul. 33, 137–149. doi: 10.3389/fpls.2017.00510

[ref69] Kubi H. A. A. Khan M. A. Adhikari A. Imran M. Kang S. M. Hamayun M.. (2021). Silicon and plant growth-promoting rhizobacteria *Pseudomonas psychrotolerans* CS51 mitigates salt stress in *Zea mays* L. Agriculture 11:272. doi: 10.3390/agriculture11030272

[ref70] Kumar R. R. Rai G. K. Kota S. Watts A. Sakhare A. Kumar S.. (2022). Fascinating dynamics of silicon in alleviation of heat stress induced oxidative damage in plants. Plant Growth Regul. 100, 321–335. doi: 10.1007/s10725-022-00879-w

[ref330] Lata R. R. Chowdhury S. Gond S. K. White J. F. (2018). Induction of abiotic stress tolerance in plants by endophytic microbes. Lett. Appl. Microbiol, 66, 268–276. doi: 10.1111/lam.12855 29359344

[ref71] Lei G. Zhang H. Y. Wang Z. H. Wei L. X. Fu P. Song J. B.. (2018). High night time temperature induces antioxidant molecule perturbations in heat-sensitive and heat-tolerant coisogenic rice (*Oryza sativa*) strains. J. Agric. Food Chem. 66, 12131–12140. doi: 10.1021/acs.jafc.8b04425, PMID: 30362740

[ref72] Li N. Euring D. Cha J. Y. Lin Z. Lu M. Huang L. J.. (2021). Plant hormone-mediated regulation of heat tolerance in response to global climate change. Front. Plant Sci. 11:627969. doi: 10.3389/fpls.2020.627969 33643337 PMC7905216

[ref73] Liang Y. C. Wong J. W. C. Wei L. (2005). Silicon-mediated enhancement of cadmium tolerance in maize (*Zea mays* L.) grown in cadmium contaminated soil. Chemosphere 58, 475–483. doi: 10.1016/j.chemosphere.2004.09.034, PMID: 15620739

[ref74] Liu J. J. Lin S. H. Xu P. L. Wang X. J. Bai J. G. (2009). Effects of exogenous silicon on the activities of antioxidant enzymes and lipid peroxidation in chilling-stressed cucumber leaves. Agric. Sci. China 8, 1075–1086. doi: 10.1016/S1671-2927(08)60315-6

[ref75] Liu P. Yin L. Wang S. Zhang M. Deng X. Zhang S.. (2015). Enhanced root hydraulic conductance by aquaporin regulation accounts for silicon alleviated salt-induced osmotic stress in *Sorghum bicolor* L. Environ. Exp. Bot. 111, 42–51. doi: 10.1016/j.envexpbot.2014.10.006

[ref76] Livak K. J. Schmittgen T. D. (2001). Analysis of relative gene expression data using real-time quantitative PCR and the 2−ΔΔCT method. Methods 25, 402–408. doi: 10.1006/meth.2001.1262 11846609

[ref77] Lowry O. H. Rose Brough N. J. Farr A. L. Randall R. J. (1951). Protein measurement with the folin phenol reagent. J. Biol. Chem. 193, 265–275. doi: 10.1016/S0021-9258(19)52451-6 14907713

[ref78] Ma J. F. Miyake Y. Takahashi E. (2001). Silicon a beneficial element for crop plants. Stud. Plant Sci. 8, 17–39. doi: 10.1016/S0928-3420(01)80006-9

[ref79] Ma J. F. Nishimura K. Takahashi E. (1989). Effect of silicon on the growth of rice plant at different growth stages. Soil Sci. Plant Nutr. 35, 347–356. doi: 10.1080/00380768.1989.10434768

[ref80] Ma J. F. Yamaji N. Mitani N. Tamai K. Konishi S. Fujiwara T. (2007). An efflux transporter of silicon in rice. Nature 12, 209–212. doi: 10.1038/nature05964 17625566

[ref81] Ma J. F. Yamaji N. Mitani-Ueno N. (2011). Transport of silicon from roots to panicles in plant. Proc. Jpn. Acad. Ser. 87, 377–385. doi: 10.2183/pjab.87.377, PMID: 21785256 PMC3171283

[ref82] Madhuri N. (2018). Addressing India's food security-is phosphorus the missing link? Econ. Polit. Wkly. 53, 17–19.

[ref83] Maestri E. Klueva N. Perrota C. Gulli M. Nguyen H. T. Marmiroli N. (2002). Molecular genetics of heat tolerance and heat shock proteins in cereals. Plant Mol. Biol. 48, 667–681. doi: 10.1023/a:1014826730024 11999842

[ref84] Mahapatra S. S. (2017). Evaluation of *Gluconacetobacter diazotrophicus* and bacillus subtilis for enhancing water deficit stress tolerance in rice (*Oryza sativa* L.) M.Sc. thesis. Available at: https://krishikosh.egranth.ac.in/handle/1/5810031593.

[ref85] Mahdieh M. Habibollahi N. Amirjani M. R. Abnosi M. H. Ghorbanpour M. (2015). Exogenous silicon nutrition ameliorates salt-induced stress by improving growth and efficiency of PSII in *Oryza sativa* L. cultivars. J. Soil Sci. Plant Nutr. 15, 1050–1060. doi: 10.4067/S0718-95162015005000073

[ref86] Mahmood S. Daur I. Al-Solaimani S. G. Ahmad S. Madkour M. H. Yasir M.. (2016). Plant growth promoting rhizobacteria and silicon synergistically enhance salinity tolerance of mung bean. Front. Plant Sci. 7:876. doi: 10.3389/fpls.2016.00876, PMID: 27379151 PMC4911404

[ref87] Mahmood S. Daur I. Yasir M. Waqas M. Hirt H. (2022). Synergistic practicing of rhizobacteria and silicon improve salt tolerance: implications from boosted oxidative metabolism, nutrient uptake, growth and grain yield in mung bean. Plants 11:1980. doi: 10.3390/plants11151980, PMID: 35956457 PMC9370704

[ref88] Marxen A. Klotzbücher T. Jahn R. Kaiser K. Nguyen V. S. Schmidt A.. (2016). Interaction between silicon cycling and straw decomposition in a silicon deficient rice production system. Plant Soil 398, 153–163. doi: 10.1007/s11104-015-2645-8

[ref89] Meena H. Ahmed A. Prakash P. (2015). Amelioration of heat stress in wheat, *Triticum aestivum* by PGPR (*Pseudomonas aeruginosa* strain 2CpS1). Biosci. Biotechnol. Res. Commun. 10, 171–174. doi: 10.3390/microorganisms10071286, PMID: 35889005 PMC9319882

[ref90] Meena V. D. Dotaniya M. L. Coumar V. Rajendiran S. Kundu S. Rao A. S. (2014). A case for silicon fertilization to improve crop yields in tropical soils. Proc. Natl. Acad. Sci. India Sec. B Biol. Sci. 84, 505–518. doi: 10.1007/s40011-013-0270-y

[ref92] Mittler R. Zandalinas S. I. Fichman Y. Van Breusegem F. (2022). Reactive oxygen species signalling in plant stress responses. Nat. Rev. Mol. Cell Biol. 23, 663–679. doi: 10.1038/s41580-022-00499-2 35760900

[ref93] Mostofa M. G. Rahman M. M. Ansary M. M. U. Keya S. S. Abdelrahman M. Miah M. G.. (2021). Silicon in mitigation of abiotic stress-induced oxidative damage in plants. Crit. Rev. Biotechnol. 41, 918–934. doi: 10.1080/07388551.2021.1892582, PMID: 33784900

[ref94] Muneer S. Park Y. G. Kim S. Jeong B. R. (2017). Foliar or subirrigation silicon supply mitigates high temperature stress in strawberry by maintaining photosynthetic and stress-responsive proteins. J. Plant Growth Regul. 36, 836–845. doi: 10.1007/s00344-017-9687-5

[ref95] Nakano Y. Asada K. (1981). Hydrogen peroxide is scavenged by ascorbate-specific peroxidase in spinach chloroplasts. Plant Cell Physiol. 22, 867–880. doi: 10.1093/oxfordjournals.pcp.a076232

[ref96] Navarro-Torre S. Rodríguez-Llorente I. D. Pajuelo E. Mateos-Naranjo E. Redondo-Gómez S. Mesa-Marín J.. (2023). “Role of bacterial endophytes in plant stress tolerance: Current research and future outlook,” in Microbial endophytes and plant growth, eds. M. K. Solanki et al. (Academic Press, 125 London Wall, London EC2Y 5AS, United Kingdom) 35–49.

[ref97] Nayyar H. Kaur R. Kaur S. Singh R. (2014). γ-Aminobutyric acid (GABA) imparts partial protection from heat stress injury to rice seedlings by improving leaf turgor and up regulating osmoprotectants and antioxidants. J. Plant Growth Regul. 33, 408–419. doi: 10.1007/s00344-013-9389-6

[ref98] Orozco-Mosqueda M. del Carmen G. S. Bernard R. G. (2023). Recent advances in the bacterial phytohormone modulation of plant growth. Plant 12:606. doi: 10.3390/plants12030606, PMID: 36771689 PMC9921776

[ref99] Pati S. Pal B. Badole S. Hazra G. C. Mandal B. (2016). Effect of silicon fertilization on growth, yield, and nutrient uptake of rice. Commun. Soil Sci. Plant Anal. 47, 284–290. doi: 10.1080/00103624.2015.1122797

[ref100] Phule A. S. Barbadikar K. M. Madhav M. S. Senguttuvel P. Babu M. B. B. P. Ananda K. P. (2018). Genes encoding membrane proteins showed stable expression in rice under aerobic condition: novel set of reference genes for expression studies. 3 Biotech 8:383. doi: 10.1007/s13205-018-1406-9, PMID: 30148033 PMC6107477

[ref101] Phule A. S. Barbadikar K. M. Maganti S. M. Senguttuvel P. Subrahmanyam D. Prasad Babu M. B. B.. (2019). RNA-seq reveals the involvement of key genes for aerobic adaptation in rice. Sci. Rep. 9:5235. doi: 10.1038/s41598-019-41703-2, PMID: 30918284 PMC6437204

[ref102] Pour-Aboughadareh A. Yousefian M. Moradkhani H. Moghaddam Vahed M. Poczai P. Siddique K. H. M. (2019). iPASTIC: an online toolkit to estimate plant abiotic stress indices. Appl. Plant Sci. 7:e11278. doi: 10.1002/aps3.11278, PMID: 31346510 PMC6636621

[ref103] Rahman M. F. Ghosal A. Alam M. F. Kabir A. H. (2017). Remediation of cadmium toxicity in field peas (*Pisum sativum* L.) through exogenous silicon. Ecotoxicol. Environ. Saf. 135, 165–172. doi: 10.1016/j.ecoenv.2016.09.019, PMID: 27736676

[ref104] Rao B. Gao L. Dai H. Hong Z. Xie H. (2019). An efficient and sustainable approach for preparing silicon fertilizer by using crystalline silica from ore. JOM 71, 3915–3922. doi: 10.1007/s11837-019-03630-5

[ref105] Rao G. B. Susmitha P. (2017). Silicon uptake, transportation and accumulation in rice. J. Pharmacogn. Phytochem. 6, 290–293.

[ref320] Raturi G. A. Sharma Y. Rana V. Thakral V. Myaka B. Salvi P.. (2021). Exploration of silicate solubilizing bacteria for sustainable agriculture and silicon biogeochemical cycle. Plant Physiol Biochem 166, 827–838. doi: 10.1016/j.plaphy.2021.06.039, PMID: 34225007

[ref106] Raza A. (2020). Metabolomics: a systems biology approach for enhancing heat stress tolerance in plants. Plant Cell Rep. 41, 741–763. doi: 10.1007/s00299-020-02635-8, PMID: 33251564

[ref107] Raza T. Abbas M. Imran S. Khan M. Y. Rebi A. Rafie-Rad Z.. (2023). Impact of silicon on plant nutrition and significance of silicon mobilizing bacteria in agronomic practices. Silicon. doi: 10.1007/s12633-023-02302-z

[ref108] Raza A. Tabassum J. Kudapa H. Varshney R. K. (2021). Can omics deliver temperature resilient ready-to-grow crops? Crit. Rev. Biotechnol. 41, 1209–1232. doi: 10.1080/07388551.2021.1898332, PMID: 33827346

[ref109] Rizwan M. Ali S. Ibrahim M. Farid M. Adrees M. Bharwana S. A.. (2015). Mechanisms of silicon-mediated alleviation of drought and salt stress in plants: a review. Environ. Sci. Pollut. Res. 22, 15416–15431. doi: 10.1007/s11356-015-5305-x, PMID: 26335528

[ref390] Roubeix V. Becquevort S. Lancelot C. (2008). Influence of bacteria and salinity on diatom biogenic silica dissolution in estuarine systems. Biogeochemistry 88, 47–62.

[ref110] Roy C. A. Roy S. K. Trivedi P. Choi J. Cho K. Yun S. H.. (2022). Label-free proteomics approach reveals candidate proteins in rice (*Oryza sativa* L.) important for ACC deaminase producing bacteria-mediated tolerance against salt stress. Environ. Microbiol. 24, 3612–3624. doi: 10.1111/1462-2920.15937, PMID: 35191581

[ref370] Saeed A. I. Sharov V. White J. Li J. Liang W. Bhagabati N. (2003). TM4: a free, open-source system for microarray data management and analysis. Biotechniques 34(2), 374–378. http://www.tigr.org/software/tm4/menu/TM4_Biotechniques_2003.pdf 12613259 10.2144/03342mt01

[ref111] Saha G. Mostofa M. G. Rahman M. M. Tran L. S. P. (2021). Silicon-mediated heat tolerance in higher plants: a molecular outlook. Plant Physiol. Biochem. 166, 341–347. doi: 10.1016/j.plaphy.2021.05.051, PMID: 34147726

[ref112] Sandhya V. Ali S. K. Z. Minakshi G. Gopal R. Venkateswarlu B. (2009). Alleviation of drought stress effects in sunflower seedlings by the exopolysaccharides producing *Pseudomonas putida* strain GAP-P45. Biol. Fertil. Soils 46, 17–26. doi: 10.1007/s00374-009-0401-z

[ref113] Sandhya K. Prakash N. B. (2019). Bioavailability of silicon from different sources and its effect on the yield of rice in acidic, neutral, and alkaline soils of Karnataka, South India. Commun. Soil Sci. Plant Anal. 50, 295–306. doi: 10.1080/00103624.2018.1563096

[ref114] Sarapat S. Longtonglang A. Umnajkitikorn K. Girdthai T. Boonkerd N. Tittabutr P.. (2020). Application of rice endophytic Bradyrhizobium strain SUTN9-2 containing modified ACC deaminase to rice cultivation under water deficit conditions. J. Plant Interact. 15, 322–334. doi: 10.1080/17429145.2020.1824028

[ref115] Sarkar A. Pramanik K. Mitra S. Soren T. Maiti T. K. (2018). Enhancement of growth and salt tolerance of rice seedlings by ACC deaminase-producing *Burkholderia* sp. MTCC 12259. J. Plant Physiol. 231, 434–442. doi: 10.1016/j.jplph.2018.10.010, PMID: 30414570

[ref116] Sarsu F.. (2018). “Screening Protocols for Heat Tolerance in Rice at the Seedling and Reproductive Stages,” in Pre-Field Screening Protocols for Heat-Tolerant Mutants in Rice, eds F. Sarsu et al. (Berlin: Springer) 9–24. doi: 10.1007/978-3-319-77338-4_2

[ref340] Shaffique S. Khan M. A. Wani S. H. Pande A. Imran M. Kang S. M.. (2022). A review on the role of endophytes and plant growth promoting rhizobacteria in mitigating heat stress in plants. Microorganisms 10, 1286. doi: 10.3390/microorganisms10071286, PMID: 35889005 PMC9319882

[ref117] Sharma L. Priya M. Kaushal N. Bhandhari K. Chaudhary S. Dhankher O. P.. (2019). Plant growth-regulating molecules as thermoprotectants: functional relevance and prospects for improving heat tolerance in food crops. J. Exp. Bot. 71, 569–594. doi: 10.1093/jxb/erz333, PMID: 31328236

[ref118] Shekhawat K. Almeida-Trapp M. Garcia-Ramirez G. X. Hirt H. (2022). Beat the heat: plant-and microbe-mediated strategies for crop thermotolerance. Trends Plant Sci. 27, 802–813. doi: 10.1016/j.tplants.2022.02.008, PMID: 35331665

[ref119] Shi W. Xiao G. Struik P. C. Jagadish K. S. Yin X. (2017). Quantifying source-sink relationships of rice under high night-time temperature combined with two nitrogen levels. Field Crops Res. 202, 36–46. doi: 10.1016/j.fcr.2016.05.013

[ref120] Silva R. Filgueiras L. Santos B. Coelho M. Silva M. Estrada-Bonilla G.. (2020). *Gluconacetobacter diazotrophicus* changes the molecular mechanisms of root development in *Oryza sativa* L. growing under water stress. Int. J. Mol. Sci. 21:333. doi: 10.3390/ijms21010333, PMID: 31947822 PMC6981854

[ref121] Sivanesan I. Son M. Soundararajan P. Jeong B. (2014). Effect of silicon on growth and temperature stress tolerance of *Nephrolepis exaltata* ‘Corditas’. Korean J. Horticult. Sci. Technol 32, 142–148. doi: 10.7235/hort.2014.13080

[ref122] Smith I. K. Vierheller T. L. Thorne C. A. (1988). Assay of glutathione reductase in crude tissue homogenates using 5, 5-dithiobis (2-nitrobenzoic acid). Anal. Biochem. 175, 408–413. doi: 10.1016/0003-2697(88)90564-7, PMID: 3239770

[ref380] Souza R. D. Ambrosini A. Passaglia L. M. (2015). Plant growth-promoting bacteria as inoculants in agricultural soils. Genet. Mol. Biol 38, 401–409. doi: 10.1590/S1415-475738420150053 26537605 PMC4763327

[ref123] Szymanska R. Slesak I. Orzechowska A. Kruk J. (2017). Physiological and biochemical responses to high light and temperature stress in plants. Environ. Exp. Bot. 139, 165–177. doi: 10.1016/j.envexpbot.2017.05.002

[ref124] Tamai K. Ma J. F. (2008). Reexamination of silicon effects on rice growth and production under field conditions using a low silicon mutant. Plant Soil 307, 21–27. doi: 10.1007/s11104-008-9571-y

[ref125] Tapera T. Akhtar S. Ahmad Z. Ejah W. Anjum S. Ahmad T.. (2018). Physiological responses of wheat to drought stress and its mitigation approaches. Acta Physiol. Plant. 40:80. doi: 10.1007/s11738-018-2651-6

[ref126] Tayade R. Ghimire A. Khan W. Lay L. Attipoe J. Q. Kim Y. (2022). Silicon as a smart fertilizer for sustainability and crop improvement. Biomol. Ther. 12:1027. doi: 10.3390/biom12081027, PMID: 35892337 PMC9332292

[ref127] Thakur M. P. van Der Putten W. H. Apon F. Angelini E. Vres B. Geisen S. (2021). Resilience of rhizosphere microbial predators and their prey communities after an extreme heat event. Funct. Ecol. 35, 216–225. doi: 10.1111/1365-2435.13696

[ref128] Tiwari S. Lata C. Chauhan P. S. Nautiyal C. S. (2016). *Pseudomonas putida* attunes morpho-physiological, biochemical and molecular responses in *Cicer arietinum* L. during drought stress and recovery. Plant Physiol. Biochem. 99, 108–117. doi: 10.1016/j.plaphy.2015.11.001, PMID: 26744996

[ref129] Tiwari Y. K. Yadav S. K. (2019). High temperature stress tolerance in maize (*Zea mays* L.): physiological and molecular mechanisms. J Plant. Biol. 62, 93–102. doi: 10.1007/s12374-018-0350-x

[ref130] Tripathi D. K. Singh S. Singh V. P. Prasad S. M. Dubey N. K. Chauhan D. K. (2017). Silicon nanoparticles more effectively alleviated UV-B stress than silicon in wheat (*Triticum aestivum*) seedlings. Plant Physiol. Biochem. 110, 70–81. doi: 10.1016/j.plaphy.2016.06.026, PMID: 27470120

[ref131] Tsukanova K. A. Chebotar V. Meyer J. J. M. Bibikova T. N. (2017). Effect of plant growth-promoting rhizobacteria on plant hormone homeostasis. S. Afr. J. Bot. 113, 91–102. doi: 10.1016/j.sajb.2017.07.007

[ref132] Ul Hassan M. Rasool T. Iqbal C. Arshad A. Abrar M. Abrar M. M.. (2021). Linking plants functioning to adaptive responses under heat stress conditions: a mechanistic review. J. Plant Growth Regul. 41, 2596–2613. doi: 10.1007/s00344-021-10493-1

[ref350] Umesh D.K.R. Madan P. Divya S. Singh R. K. Sini T. (2016). Identifying veritable sources of heat tolerance in rice (*Oryza sativa* L.). Int. J. Agric. Sci. 8(53), 2743–2746.

[ref133] Verma K. K. Liu X. H. Wu K. C. Singh R. K. Song Q. Q. Malviya M. K.. (2019). The impact of silicon on photosynthetic and biochemical responses of sugarcane under different soil moisture levels. Silicon 12, 1355–1367. doi: 10.1007/s12633-019-00228-z

[ref134] Vishwakarma K. Singh V. P. Prasad S. M. Chauhan D. K. Tripathi D. K. Sharma S. (2020). Silicon and plant growth promoting rhizobacteria differentially regulate AgNP-induced toxicity in *Brassica juncea*: implication of nitric oxide. J. Hazard. Mater. 390:121806. doi: 10.1016/j.jhazmat.2019.121806, PMID: 32058900

[ref135] Wang W. Wu Z. He Y. Huang Y. Li X. Ye B. C. (2018). Plant growth promotion and alleviation of salinity stress in *Capsicum annuum* L. by Bacillus isolated from saline soil in Xinjiang. Ecotoxicol. Environ. Saf. 164, 520–529. doi: 10.1016/j.ecoenv.2018.08.070, PMID: 30149350

[ref136] Xu Y. F. Chu C. C. Yao S. G. (2021). The impact of high-temperature stress on rice: challenges and solutions. Crop J. 9, 963–976. doi: 10.1016/j.cj.2021.02.011

[ref137] Yamaji N. Mitatni N. Ma J. F. (2008). A transporter regulating silicon distribution in rice shoots. Plant Cell 20, 1381–1389. doi: 10.1105/tpc.108.059311, PMID: 18515498 PMC2438455

[ref138] Yamaji N. Sakurai G. Mitani-Ueno N. Ma J. F. (2015). Orchestration of three transporters and distinct vascular structures in node for intervascular transfer of silicon in rice. Proc. Natl. Acad. Sci. 112, 11401–11406. doi: 10.1073/pnas.1508987112, PMID: 26283388 PMC4568664

[ref139] Yin L. Wang S. Tanaka K. Fujihara S. Itai A. Den X.. (2016). Silicon-mediated changes in polyamines participate in silicon-induced salt tolerance in *Sorghum bicolor* L. Plant Cell Environ. 39, 245–258. doi: 10.1111/pce.12521 25753986

[ref140] Yoshida S. Forno D. A. Cock J. H. Gomez K. A. (1972). Laboratory manual for physiological studies of Rice. International Rice Research Institute: Manila.

[ref141] Younis A. A. Khattab H. Emam M. M. (2020). Impacts of silicon and silicon nanoparticles on leaf ultrastructure and TaPIP1 and TaNIP2 gene expressions in heat stressed wheat seedlings. Biol. Plant 64, 343–352. doi: 10.32615/bp.2020.030

[ref142] Zafar S. A. Hameed A. Nawaz M. A. Wei M. A. Noor M. A. Hussain M.. (2018). Mechanisms and molecular approaches for heat tolerance in rice (*Oryza sativa* L.) under climate change scenario. J. Integrative Agric. 17, 726–738. doi: 10.1016/S2095-3119(17)61718-0

[ref143] Zargar S. M. Mahajan R. Bhat J. A. Nazir M. Deshmukh R. (2019). Role of silicon in plant stress tolerance: opportunities to achieve a sustainable cropping system. 3 Biotech 9:73. doi: 10.1007/s13205-019-1613-z, PMID: 30800584 PMC6368905

[ref144] Zellener W. Tubana B. Rodrigues F. A. Datnoff L. E. (2021). Silicon’s role in plant stress reduction and why this element is not used routinely for managing plant health. Plant Dis. 105, 2033–2049. doi: 10.1094/PDIS-08-20-1797-FE, PMID: 33455444

[ref145] Zhang J. Han C. Liu Z. (2009). Absorption spectrum estimating rice chlorophyll concentration: preliminary investigations. J. Plant Breed. Crop Sci. 1, 223–229. doi: 10.5897/JPBCS.9000004

[ref146] Zhao C. Liu B. Piao S. Wang X. Lobell D. B. Huang Y.. (2017). Temperature increase reduces global yields of major crops in four independent estimates. Proc. Natl. Acad. Sci. 114, 9326–9331. doi: 10.1073/pnas.1701762114, PMID: 28811375 PMC5584412

[ref147] Zhou R. Yu X. Ottosen C. O. Rosenqvist E. Zhao L. Wang Y.. (2017). Drought stress had a predominant effect over heat stress on three tomato cultivars subjected to combined stress. BMC Plant Biol. 17, 24–13. doi: 10.1186/s12870-017-0974-x, PMID: 28122507 PMC5264292

[ref148] Zhu Y. Gong H. (2014). Beneficial effects of silicon on salt and drought tolerance in plants. Agron. Sustain. Dev. 34, 455–472. doi: 10.1007/s13593-013-0194-1

