# Supplementary material for: Silicate solubilizing and plant growth promoting bacteria interact with biogenic silica to impart heat stress tolerance in rice by modulating physiology and gene expression
Source: Front Microbiol. 2023 Jul 13;14:1168415. doi: 10.3389/fmicb.2023.1168415 (PMC10374332; doi:10.3389/fmicb.2023.1168415)
Supplement: Supplementary file 1 [file Data_Sheet_1.docx]

**Supplementary Material**


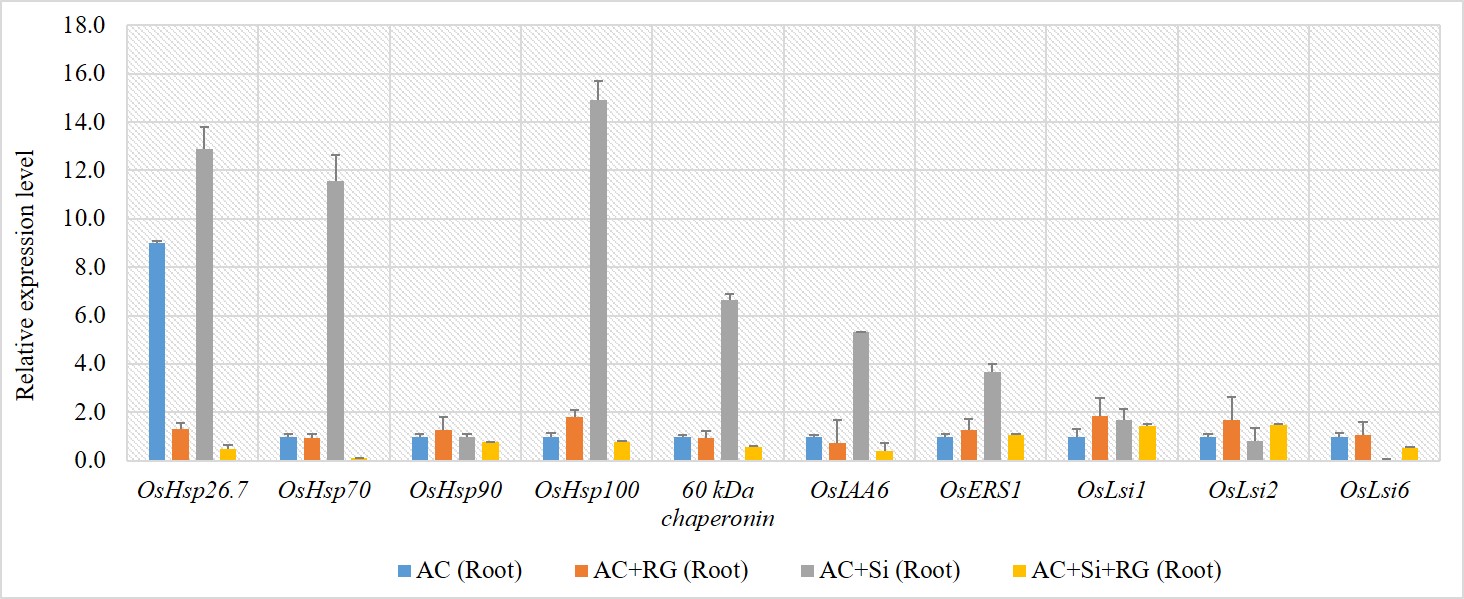


**Supplementary Figure S1**. Relative expression of HSPs, hormone-related and silicon transporter genes in root tissue of rice seedlings under ambient conditions. Data were normalized by using *UBQ6* as an internal control gene. The error bar on the top represents a mean ± SD (three technical replicates/ biological replicates).


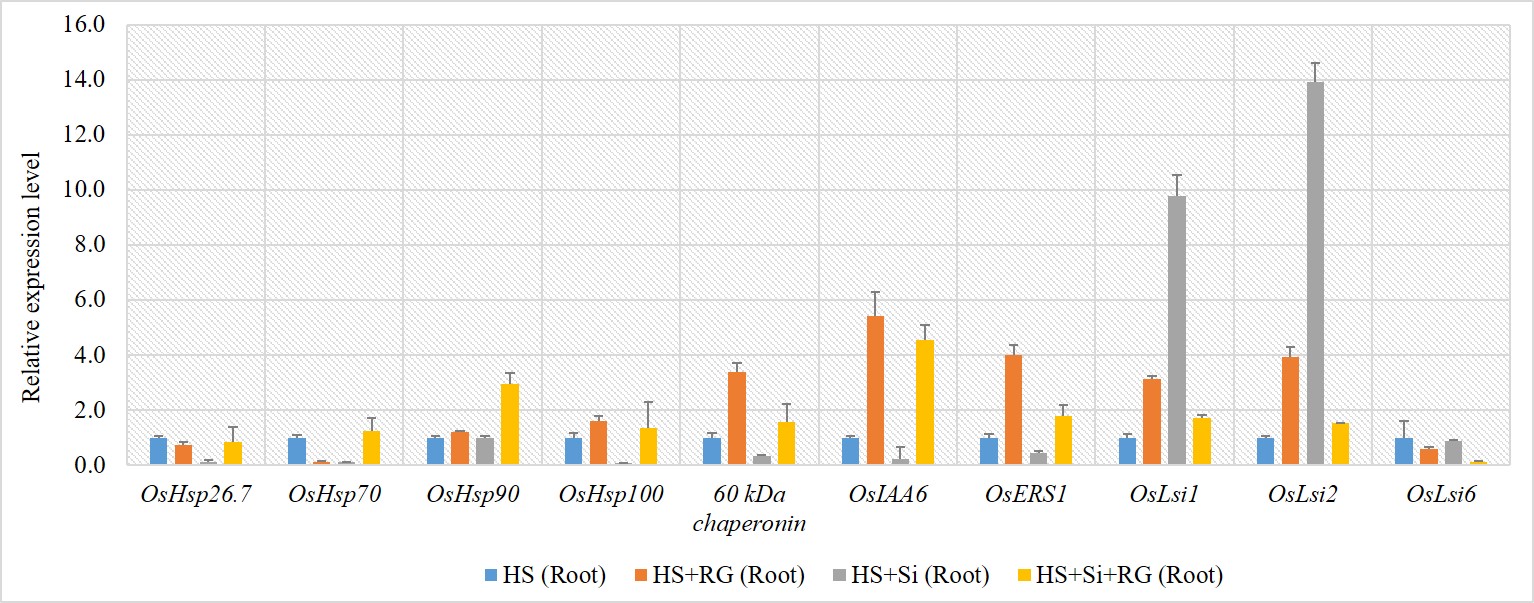


**Supplementary Figure S2**. Relative expression of HSPs, hormone-related and silicon transporter genes in the root tissue of rice seedlings under heat stress conditions**.** Data were normalized by using *UBQ6* as an internal control gene. The error bar on the top represents a mean ± SD (three technical replicates/ biological replicates).


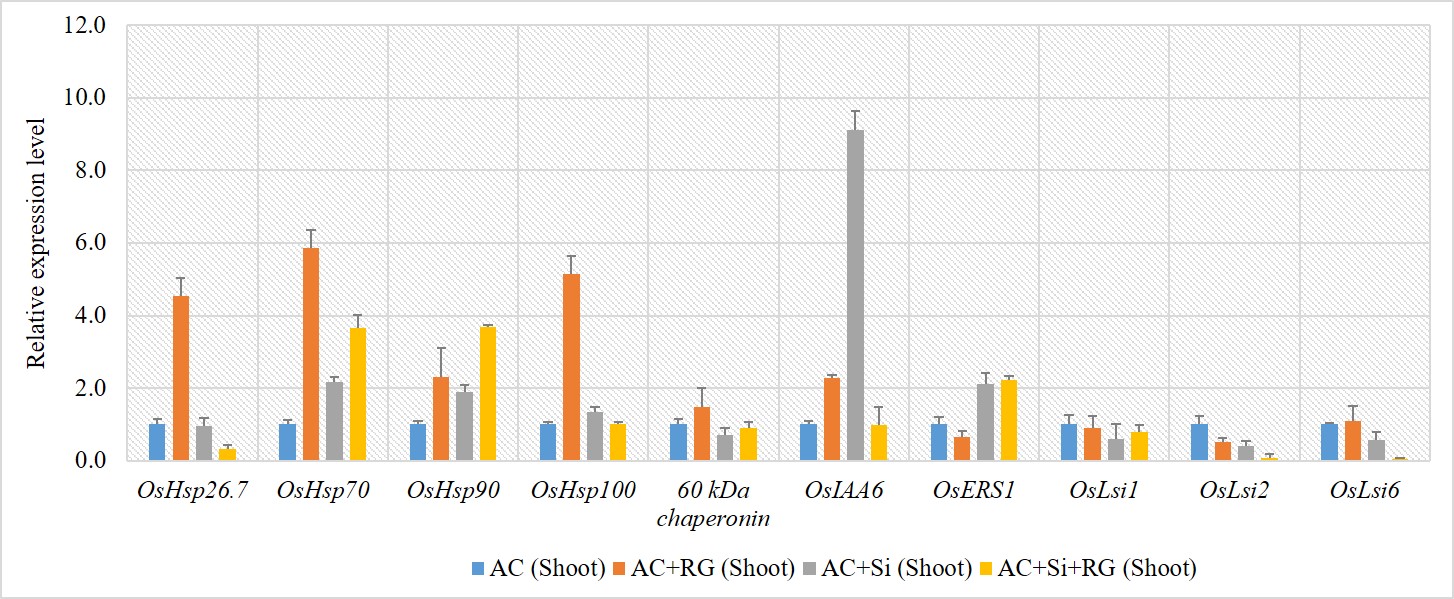


**Supplementary Figure S3**. Relative expression of HSPs, hormone-related and silicon transporter genes in shoot tissue of rice seedlings under ambient conditions. Data were normalized by using *UBQ6* as an internal control gene. The error bar on the top represents a mean ± SD (three technical replicates/ biological replicates).


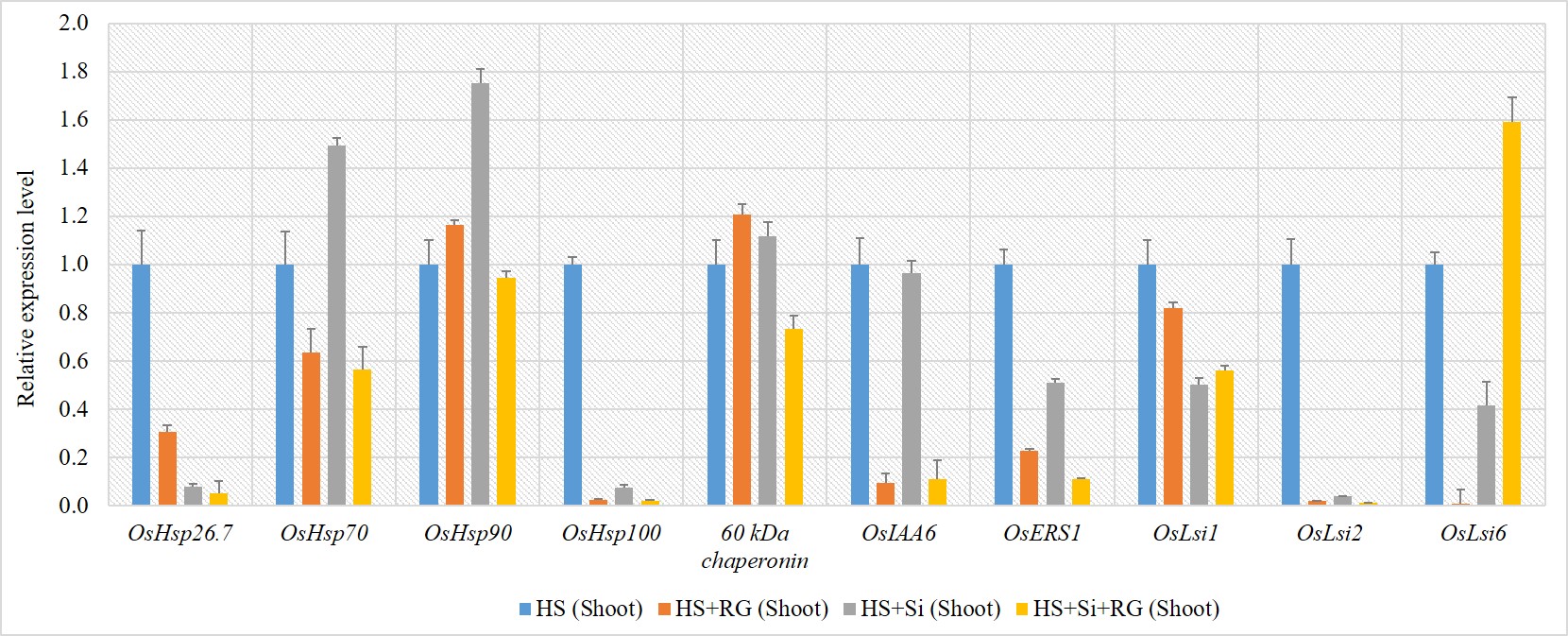


**Supplementary Figure S4**. Relative expression of HSPs, hormone-related and silicon transporter genes in shoot tissue of rice seedlings at heat stress conditions. Data were normalized by using *UBQ6* as an internal control gene. The error bar on the top represents a mean ± SD (three technical replicates/ biological replicates).

**Supplementary Table S1.** List of primers used in the study for quantitative real-time PCR analysis.

| **S.No** | **Gene symbol** | **Gene description** | **Locus information (MSU)** | **Primer sequence 5′-3′**  **(forward /reverse)** |
| --- | --- | --- | --- | --- |
| **Heat shock proteins** | | | | |
| 1 | *OsHsp 26.7* | Heat shock protein 26.7 | LOC_Os03g14180 | TCAGCGAACGAGCGAATGAATGG |
|  |  |  |  | AGGCATGAGGTGATGAAGCAACC |
| 2 | *OsHSP 70* | Heat shock protein 70 | LOC_Os01g62290 | [TCGATGCCAAGCGACTGATAGG](about:blank) |
|  |  |  |  | [GCACCACAATCATAGGCTTATCGC](about:blank) |
| 3 | *OsHsp 90* | Heat shock protein 90 | LOC_Os08g38086.3 | [AGCCTTTACAGCCACAAGGAAG](about:blank) |
|  |  |  |  | [ATGCGTCACTCGCATTACTCAC](about:blank) |
| 4 | *OsHsp 100* | Heat shock protein 100 | LOC_Os03g32560 | GTTGCATCGCCGCTGATTGATG |
|  |  |  |  | TATTTCGAAGCGGACGTCTCAACG |
| 5 | *60 kDa Chaperonin* | 60 kDa Chaperonin | LOC_Os10g32550 | [TTGGCTATGATGCGGCTAAAGGTG](about:blank) |
|  |  |  |  | [ACGATGATGGATTCCGTGGTTGTC](about:blank) |
| **Auxin and ethylene response factors** | | | | |
| 6 | *OsIAA6* | Auxin-response protein | LOC_Os01g53880 | CACCATGGAAGAAGGGTCCAAAA |
|  |  |  |  | TTAGACCCTAGCAGTAGCTCCA |
| 7 | *OsERS1* | Ethylene-response factor1 | LOC_Os04g46220 | GAAAGGTCAGGCTTCTCTGAAATC |
|  |  |  |  | ATGCCGTCGATCAATTTACAGTAG |
| **Silicon transporters** | | | | |
| 8 | *OsLsi1* | Low Silicon Rice 1 | LOC_Os02g51110 | CGGTGGATGTGATCGGAACCA |
|  |  |  |  | CGTCGAACTTGTTGCTCGCCA |
| 9 | *OsLsi2* | Low Silicon Rice 2 | LOC_Os03g01700 | ATCTGGGACTTCATGGCCC |
|  |  |  |  | ACGTTTGATGCGAGGTTGG |
| 10 | *OsLsi6* | Low Silicon Rice 6 | LOC_Os06g12310 | GAGTTCGACAACGTCTAATCGC |
|  |  |  |  | AGTACACGGTACATGTATACACG |

**Supplementary Table S2**. Correlation analyses between different morphological, physiological and biochemical (antioxidant enzymatic activities) traits under ambient and heat stress conditions.

|  | **Root biomass** | **Si content in root** | **Shoot biomass** | **Si content in the shoot** | **Chlorophyll content** | **Electrolyte leakage** | **Relative water content** | **Superoxidase dismutase** | **Catalase** | **Ascorbate peroxidase** | **Glutathione reductase** |
| --- | --- | --- | --- | --- | --- | --- | --- | --- | --- | --- | --- |
| **Root biomass** | 1.00 |  |  |  |  |  |  |  |  |  |  |
| **Si content in root** | **0.85** | 1.00 |  |  |  |  |  |  |  |  |  |
| **Shoot biomass** | **0.82** | **0.97** | 1.00 |  |  |  |  |  |  |  |  |
| **Si content in the shoot** | 0.75 | **0.93** | **0.84** | 1.00 |  |  |  |  |  |  |  |
| **Chlorophyll content** | **0.92** | **0.86** | 0.77 | **0.86** | 1.00 |  |  |  |  |  |  |
| **Electrolyte leakage** | **-0.94** | -0.81 | -0.77 | -0.67 | **-0.91** | 1.00 |  |  |  |  |  |
| **Relative water content** | **0.92** | **0.82** | 0.79 | 0.70 | **0.93** | **-0.99** | 1.00 |  |  |  |  |
| **Superoxidase dismutase** | **0.89** | 0.78 | 0.74 | 0.72 | **0.92** | **-0.93** | **0.92** | 1.00 |  |  |  |
| **Catalase** | **0.94** | **0.87** | **0.86** | 0.70 | **0.88** | **-0.96** | **0.93** | **0.91** | 1.00 |  |  |
| **Ascorbate peroxidase** | **0.86** | 0.79 | 0.79 | 0.67 | **0.85** | **-0.92** | **0.89** | **0.97** | **0.94** | 1.00 |  |
| **Glutathione reductase** | **0.95** | **0.83** | **0.81** | 0.71 | **0.92** | **-0.98** | **0.96** | **0.96** | **0.98** | **0.96** | 1.00 |

Bold values indicate p < 0.01 while non-bold values are p < 0.05

**Supplementary Table S3**. Heat stress tolerance index of seedlings under different treatments.

| **Treatment** | **Seedling biomass under ambient temperature (Yp)** | **Seedling biomass under heat stress (Ys)** | **Stress tolerance index (STI)** |
| --- | --- | --- | --- |
| HS | 0.28 | 0.19 | 0.44 |
| HS+RG (Inoculation with *Rhizobium sp. IIRR-1* and *Gluconacetobacter diazotrophicus)* | 0.33 | 0.26 | 0.71 |
| HS+Si (Application of silicates) | 0.37 | 0.24 | 0.74 |
| HS+Si+RG (application of silicates combined with *Rhizobium sp. IIRR-1* and *Gluconacetobacter* *diazotrophicus* inoculation) | 0.41 | 0.33 | 1.12 |
